# MTHFD1 controls DNA methylation in *Arabidopsis*

**DOI:** 10.1038/ncomms11640

**Published:** 2016-06-13

**Authors:** Martin Groth, Guillaume Moissiard, Markus Wirtz, Haifeng Wang, Carolina Garcia-Salinas, Perla A. Ramos-Parra, Sylvain Bischof, Suhua Feng, Shawn J. Cokus, Amala John, Danielle C. Smith, Jixian Zhai, Christopher J. Hale, Jeff A. Long, Ruediger Hell, Rocío I. Díaz de la Garza, Steven E. Jacobsen

**Affiliations:** 1 Department of Molecular, Cell, and Developmental Biology, University of California Los Angeles, Los Angeles, California 90095, USA; 2 Centre for Organismal Studies, University of Heidelberg, Heidelberg 69120, Germany; 3 Basic Forestry and Proteomics Research Center, Haixia Institute of Science and Technology (HIST), Fujian Province Key Laboratory of Plant Virology, Institute of Plant Virology, Fujian Agriculture and Forestry University, Fuzhou, Fujian 350002, China; 4 Tecnologico de Monterrey, Campus Monterrey, Ave. Eugenio Garza Sada 2501, Monterrey 64849, México; 5 Eli & Edythe Broad Center of Regenerative Medicine & Stem Cell Research, University of California Los Angeles, Los Angeles, California 90095, USA; 6 Howard Hughes Medical Institute, University of California Los Angeles, Los Angeles, California 90095, USA

## Abstract

DNA methylation is an epigenetic mechanism that has important functions in transcriptional silencing and is associated with repressive histone methylation (H3K9me). To further investigate silencing mechanisms, we screened a mutagenized *Arabidopsis thaliana* population for expression of *SDCpro-GFP*, redundantly controlled by DNA methyltransferases DRM2 and CMT3. Here, we identify the hypomorphic mutant *mthfd1-1*, carrying a mutation (R175Q) in the cytoplasmic bifunctional methylenetetrahydrofolate dehydrogenase/methenyltetrahydrofolate cyclohydrolase (MTHFD1). Decreased levels of oxidized tetrahydrofolates in *mthfd1-1* and lethality of loss-of-function demonstrate the essential enzymatic role of MTHFD1 in *Arabidopsis*. Accumulation of homocysteine and S-adenosylhomocysteine, genome-wide DNA hypomethylation, loss of H3K9me and transposon derepression indicate that S-adenosylmethionine-dependent transmethylation is inhibited in *mthfd1-1*. Comparative analysis of DNA methylation revealed that the CMT3 and CMT2 pathways involving positive feedback with H3K9me are mostly affected. Our work highlights the sensitivity of epigenetic networks to one-carbon metabolism due to their common S-adenosylmethionine-dependent transmethylation and has implications for human MTHFD1-associated diseases.

## Abstract (web summary)

DNA methylation contributes to transcriptional silencing. Here, Groth *et al.* show that mutant plants defective in MTHFD1, an enzyme involved in folate metabolism, have a DNA hypomethylation phenotype highlighting the link between one-carbon metabolism and DNA methylation, which is mediated by SAM as a common methyl donor.

## Graphical abstract

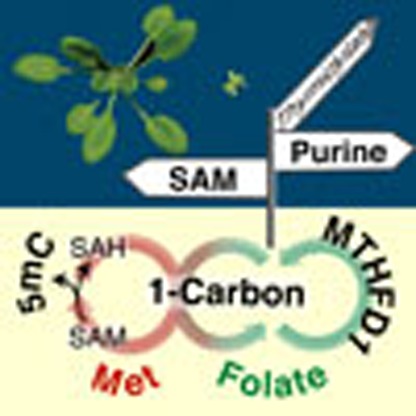

DNA methylation serves as a defense mechanism against transposable elements (TEs) and other types of repetitive DNA that can harm the genome of the organism they inhabit. DNA methylation promotes the packaging of DNA into so-called heterochromatin as to enforce a silent state, for example, by making the DNA inaccessible to transcription activators. Because of its transgenerational stability, DNA methylation is the prime example of an epigenetic mechanism. The stability is provided by feedback loops within, as well as crosstalk between, different methylation pathways, as established in *Arabidopsis thaliana* (*Arabidopsis*), where DNA methylation occurs at CG, CHG and CHH (H=A, T or C)^1^. In contrast to CHG and CHH methylation, which are exclusively involved in heterochromatin formation and transcriptional gene silencing (TGS), CG methylation also occurs over gene bodies.

DNA methylation is generated by the activity of DNA methyltransferases (DNMTs), which enzymatically transfer a methyl group from S-adenosyl methionine (SAM) to cytosine. In *Arabidopsis*, DOMAINS REARRANGED METHYLTRANS-FERASE2 (DRM2) catalyses *de novo* methylation in all sequence contexts^2^. Once established, DNA methylation at symmetric CG and CHG sequences is maintained by DNA METHYLTRANSFERASE1 (MET1) and CHROMOMETHYLASE3 (CMT3), respectively. Maintenance of CG methylation is based on the recognition of hemimethylated signatures after semiconservative DNA replication. Analogous to the recruitment of the mammalian maintenance methylase DNMT1 through UHRF1, members of the VARIANT IN METHYLATION (VIM) family bind to hemimethylated DNA with their SET and RING-associated (SRA) domains and are required for CG methylation by MET1 (refs 1, 3). In contrast, CHG methylation is maintained by a reinforcing loop between non-CG methylation and methylation of lysine 9 of histone H3 (H3K9me), which involves the histone methyltransferases KRYPTONITE/SUVH4, 5 and 6. These preferentially bind methylated non-CG sequences via their SRA domains and modify the wrapped nucleosome with H3K9me (ref. 4). In turn, CMT3 binds H3K9me through its chromo and BAH domains and catalyses the remethylation of CHG sites during replication^5^. Similarly, maintenance of CHH methylation by CMT2 also depends on SUVH4/5/6-mediated H3K9me (ref. 6).

While CMT2 and 3 mostly target transposons in the pericentromeric heterochromatin, DRM2 is mainly required for maintenance of CHH methylation and TGS in the chromosomal arms^7^. Targeting of DRM2 is mediated by the concerted action of short transcripts that are processed into 24 nt small-interfering RNAs and complementary long noncoding transcripts produced by the plant-specific RNA polymerase complexes Pol IV and Pol V, respectively. In the canonical RNA-directed DNA methylation pathway (RdDM), 24 nt RNAs are incorporated into ARGONAUT4 (AGO4) in order to match the RNA-induced silencing complexes with Pol V transcripts^8^. Subsequently, DRM2 is recruited to the target CHH sites by direct interaction with AGO4 (ref. 9). Recruitment of Pol IV to heterochromatin is also dependent on H3K9me interaction via SAWADEE HOMEODOMAIN HOMOLOGue 1 (SHH1) (ref. 10), whereas Pol V is recruited via the non-catalytic SUV39 homologues SUVH2 and 9 that bind to methylated DNA via their SRA domains^11^.

TGS is often reinforced by the synergistic action of different DNA methylation pathways. This is exemplified by the *SUPPRESSOR OF DRM2 CMT3 (SDC)* locus, which is redundantly silenced by the CMT3- and DRM2-mediated methylation of tandem repeats in the promoter region^12^. Hence, *SDC* is ectopically expressed in *drm2 cmt3* double mutants, but repressed during most of development in the single mutants, making *SDC* a powerful genetic marker of simultaneous impairment of CHG and CHH methylation pathways. We generated stable transgenic lines carrying an *SDC*_*pro*_*-GFP* fusion construct in wild-type (WT) and *cmt3* genetic background and screened M2 populations for EMS-mutants that express GFP. The identification of *microrchidia 1* (*morc1*) and *morc6* mutants from this screen was published previously^13^. Here, we identified a mutant from the WT background that carries a missense mutation in the *Arabidopsis METHYLENETETRAHYDROFOLATE DEHYDROGENASE/METHENYLTETRAHYDROFOLATE CYCLOHYDROLASE 1* (*MTHFD1*) gene. The mutation disrupts folate metabolism and leads to accumulation of homocysteine (Hcy), a hallmark of an impaired methionine (Met) cycle, whose main function is to produce SAM for transmethylation reactions and recycle the byproduct S-adenosyl homocysteine (SAH)^14^. Genome-wide loss of CHG and CHH methylation, reduced H3K9me and derepression of TEs in the *mthfd1-1* mutant indicate that the Met cycle constitutes an ‘Achilles heel' of the feedback mechanisms between DNA and histone methylation.

## Results

### The R175Q mutation in MTHFD1 leads to *SDCpro*-GFP expression

**Figure 1:**
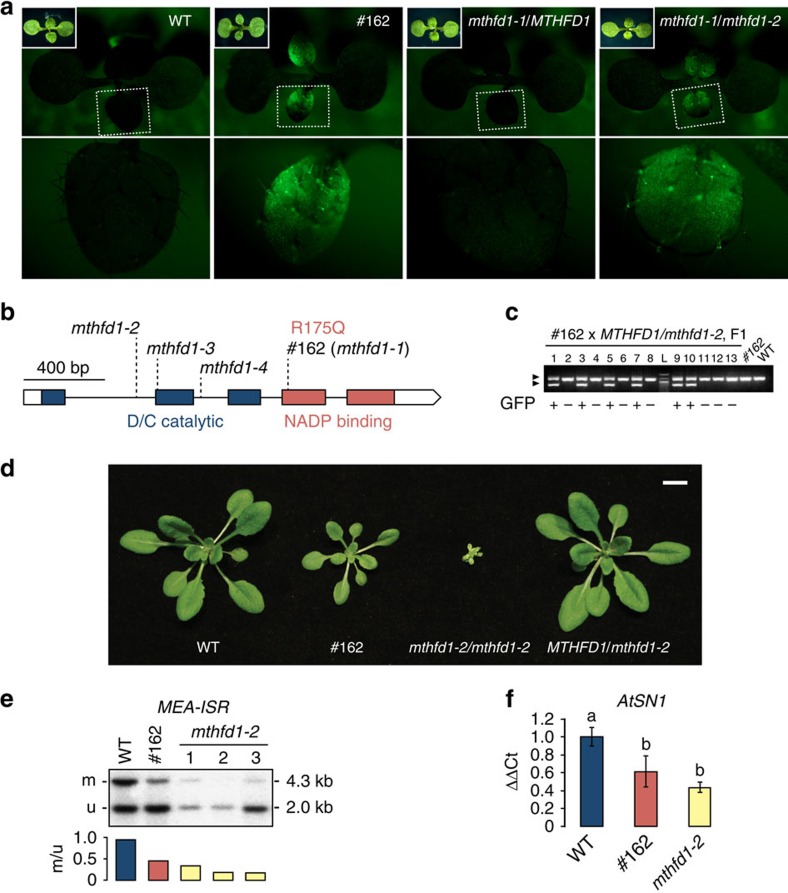
*SDCpro-GFP* expression and DNA demethylation caused by R175Q mutation in *MTHFD1*. (**a**) GFP fluorescence micrographs of WT, #162 M2, MTHFD1/*mthfd1-2* F1 and #162/*mthfd1-2* F1 seedlings. F1 are progeny of #162 M2 x *MTHFD1/mthfd1-2*. Dashed boxes indicate magnified areas shown in lower panels. Inlets show bright-field images. (**b**) Gene structure, positions of mutations and conserved domains of MTHFD1. The EMS mutation in #162 lead to a R175Q substitution of a conserved residue required for NADP binding^28^. (**c**) PCR-based genotype analysis of 13 F1 seedlings and two control samples. Arrowheads mark bands corresponding to WT/*mthfd1-1* (upper) and *mthfd1-2* (lower). The *mthfd1-2* allele co-segregates with GFP fluorescence in F1 (+: present, −: absent). L, ladder. (**d**) Habit of different genotype plants 20 days after germination. Scale bar, 10 mm. (**e**) DNA blot analysis of non-CG methylation at the *MEA-ISR* locus. Genomic DNA was digested with methylation-sensitive MspI; upper and lower bands correspond to methylated (m) and unmethylated (u) fragments, respectively. Ratios of band intensities for each lane are shown under the gel image. (**f**) Levels of non-CG methylation at the *AtSN1* locus by quantitative chop PCR analysis of genomic DNA after digestion with methylation-sensitive HaeIII relative to undigested DNA. Mean values±s.d. (*n*=3). Different letters above bars indicate significant differences between pairwise comparisons by Student's *t*-test (*P*<0.05).

Mutant #162 was identified by screening M2 seedlings of an EMS-mutagenized population of *Arabidopsis* that carried an *SDCpro-GFP* insertion event in WT background (herein after referred to as WT; Col refers to non-transgenic WT) for individuals that showed GFP fluorescence (Fig. 1a). Using deep sequencing of bulked GFP-positive F2 progeny of mutant #162 crossed with a WT plant of ecotype Landsberg *erecta* (L*er*), we confined the target region containing the causative mutation to the north end of chromosome 3 (Supplementary Fig. 1). To identify the causative mutation, mutant #162 was crossed to WT and the co-segregation of candidate EMS mutations in GFP-positive F2 progeny was analysed using dCAPS markers (Supplementary Fig. 2). A guanine to adenine transition in a gene (At3g12290) encoding a putative methylenetetrahydrofolate dehydrogenase/methenyltetrahydrofolate cyclohydrolase (MTHFD1) showed 100% co-segregation in 98 GFP-positive F2 individuals (Supplementary Fig. 2). The mutant phenotype segregated as a recessive monogenic trait (103 GFP-positive versus 368 GFP-negative, χ^2^ *P* value (3:1)=0.12). The mutation, herein after named *mthfd1-1*, leads to a predicted substitution of a conserved arginine by glutamine at residue 175 (R175Q) (Fig. 1b). To confirm that *mthfd1-1* caused the expression of *SDCpro-GFP*, a #162 M3 mutant (*mthfd1-1/mthfd1-1*) was crossed with a heterozygous plant containing a transfer DNA (T-DNA) insertion allele, *MTHFD1*/*mthfd1-2* (Fig. 1b). GFP expression in F1 progeny co-segregated with the *mthfd1-2* allele (Fig. 1a,c), confirming that *mthfd1-1* caused the expression of *SDCpro-GFP* in #162.

GFP-positive plants originating from mutant #162 had pale leaves, reduced seed set, were smaller and developed more slowly compared with WT but did not display other morphological defects (Fig. 1d and Supplementary Fig. 3a). In contrast, *mthfd1-2* homozygous mutants showed severe developmental defects, including dwarfism, pale, shortened leaves, reduced apical dominance, delayed flowering, prolonged vegetative phase and infertility (Fig. 1d and Supplementary Fig. 3a,b). Moreover, on average we only retrieved one viable homozygous *mthfd1-2* mutant out of 18 seeds from a heterozygous parent, indicating that more than 75% of homozygous *mthfd1-2* mutants died prematurely (Supplementary Table 1). Viability seemed to be affected during or after germination, because siliques from WT and heterozygous *MTHFD1*/*mthfd1-2* did not show differences in ovule and seed development (Supplementary Fig. 3a). We did not retrieve any homozygous mutants from the T-DNA alleles *mthfd1-3* or *mthfd1-4* (Fig. 1b). These results led to the conclusion that MTHFD1 is an essential gene in *Arabidopsis* and that the T-DNA insertion in the first intron in *mthfd1-2* does not completely abolish the function of *MTHFD1.* Moreover, the results indicate that R175Q in *mthfd1-1* partially impairs gene function, causing a hypomorphic phenotype that does not affect viability.

To test if DNA methylation is altered in *mthfd1* mutants, we analysed the *MEDEA INTERGENIC SUBTELOMERIC REPEAT* (*MEA-ISR*) locus using a well-established Southern blot assay^15^. In WT, approximately half of the *MEA-ISR* alleles are methylated and restriction digest with methylation-sensitive MspI produces two fragments of similar abundance. Both #162 and *mthfd1-2* mutants showed a reduction of the methylated band, and the loss of methylation was stronger in *mthfd1-2* than in #162 (Fig. 1e). We also analysed DNA methylation at *Arabidopsis thaliana SHORT INTERSPERSED ELEMENT 1* (*AtSN1*) by quantitative PCR following methylation-sensitive restriction digestion and observed similar reductions in DNA methylation (Fig. 1f). Furthermore, bisulfite (BS)-PCR analysis of the levels of methylation at CG, CHG and CHH sites in the tandem repeat region of the transgenic *SDC* promoter showed a decrease in CHG methylation of the transgenic *SDC* in #162 mutants compared with the WT reference (Supplementary Fig. 4). In summary, the DNA methylation assays confirmed that MTHFD1 is required for DNA methylation at different loci in different sequence contexts, and the R175Q amino-acid substitution leads to reduced DNA methylation. Because of the limiting amounts of tissue available from *mthfd1-2* mutants, only #162 mutants (referred to as *mthfd1-1* herein after) were analysed subsequently.

### MTHFD1 is required for epigenetic silencing

**Figure 2:**
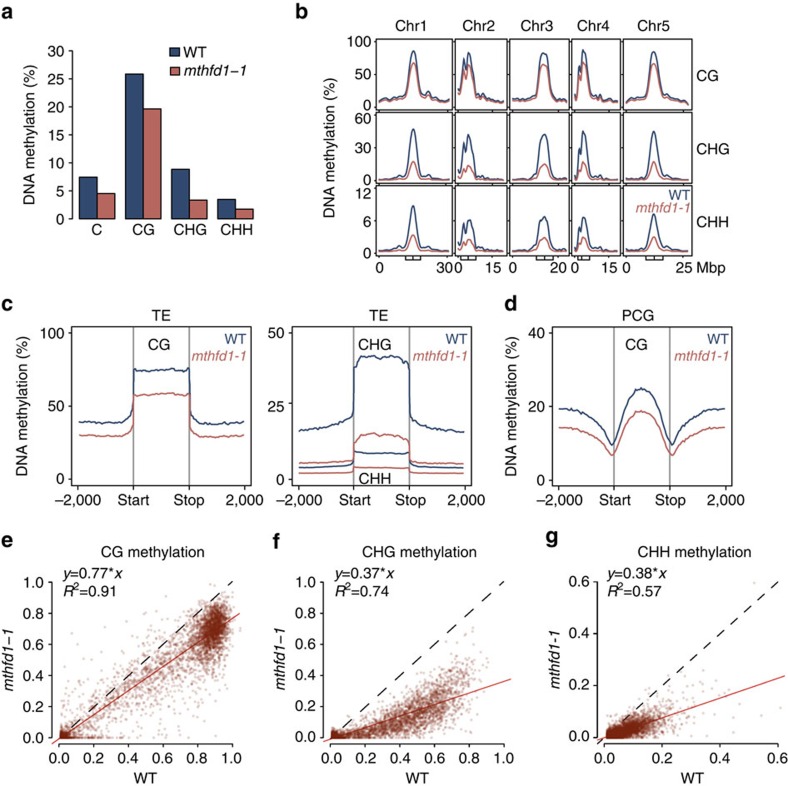
DNA methylation is globally decreased in *mthfd1-1* mutants. (**a**) Average genome-wide DNA methylation for all Cs and in individual sequence contexts (H=C, A or T). (**b**) Chromosomal distribution of fractional DNA methylation in individual sequence contexts. Boxes and vertical lines inside boxes mark pericentromeric regions and centromeres, respectively. (**c**,**d**) Average distribution of DNA methylation over TEs (**c**) and PCGs (**d**) and the flanking 2,000 bp in individual sequence contexts. (**e**–**g**) Comparison of DNA methylation levels in 5,000 random 100 bp bins with WT methylation levels >0.01 in CG (**e**), CHG (**f**) and CHH (**g**) contexts. Red line: linear regression between *mthfd1-1* and WT levels; corresponding coefficients are shown in top left corners. Dashed: identity line.

To get a general view of the DNA methylation defects in *mthfd1-1*, we analysed genome-wide DNA methylation at single-nucleotide resolution by BS-seq. The average global DNA methylation was reduced by ∼40% relative to WT (Fig. 2a). The strongest effect was observed in the CHG context, which lost 62% of DNA methylation relative to WT, followed by CHH and CG methylation with 50% and 24% decreases, respectively (Fig. 2a). DNA methylation in all sequence contexts was mostly decreased over the TE-rich pericentromeric regions, which contain most of the DNA methylation along the chromosomes (Fig. 2b). Accordingly, average methylation levels over TEs were strongly decreased in *mthfd1-1* compared with WT, especially in CHG and CHH contexts (Fig. 2c). DNA methylation was also moderately decreased over protein-coding genes (PCGs) (Fig. 2d), indicating that MTHFD1 is not only required for repressive DNA methylation at TEs but also for efficient gene body CG methylation, although to a lesser degree, compared with non-CG methylation. The different effects on DNA methylation in the different sequence contexts was also apparent when comparing WT and *mthfd1-1* CG, CHG and CHH methylation levels in randomly selected 100 bp windows of the genome with methylation thresholds >1% (in order to exclude unmethylated bins during randomization) (Fig. 2e–g). This comparison additionally shows a positive correlation of DNA methylation levels between WT and *mthfd1-1* in all three sequence contexts, which indicates that DNA methylation was decreased uniformly across the genome (Fig. 2e–g), as opposed to the DNA methylation patterns in loss-of-function DNMT mutants, which show either nearly complete loss across the sample pool (Supplementary Fig. 5a,b), or at a subset of regions (Supplementary Fig. 5c).

**Figure 3:**
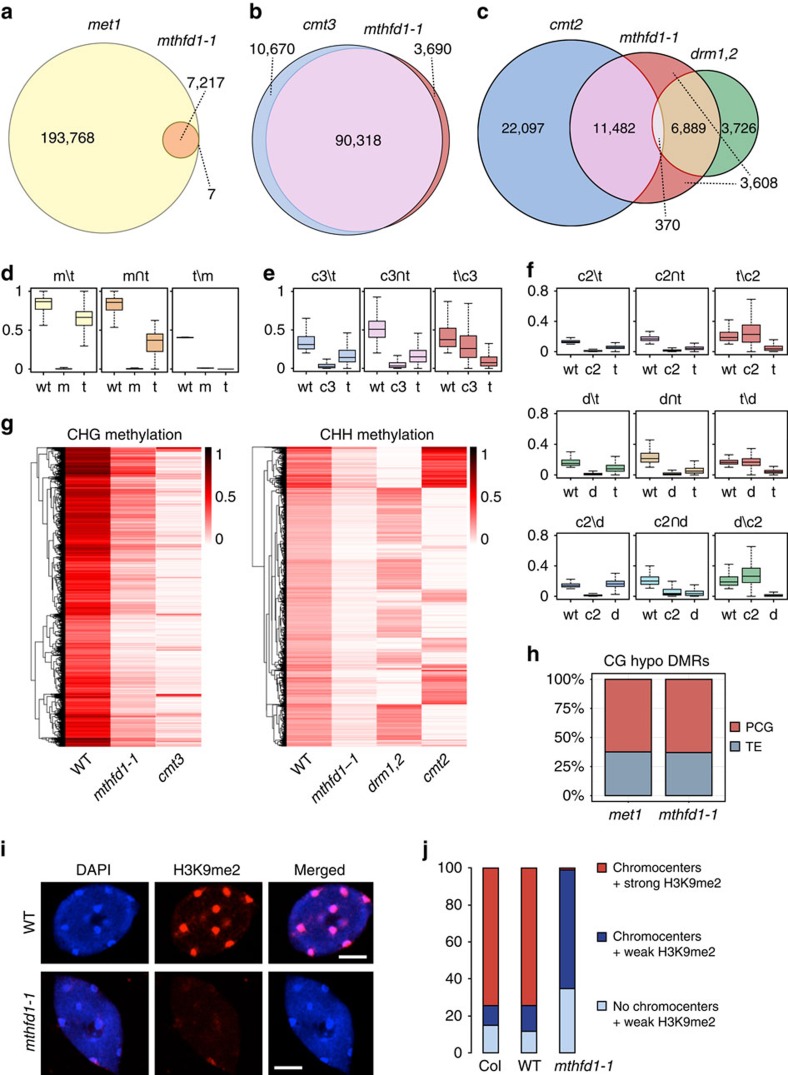
*mthfd1-1* mostly interferes with non-CG and H3K9 methylation. (**a**–**c**) Overlap between hypo-DMRs of different mutants in CG (**a**), CHG (**b**) and CHH (**c**) contexts. (**d**–**f**) CG (**d**), CHG (**e**) and CHH (**f**) methylation levels in DMR fractions corresponding to (**a**–**c**), respectively. w=wild-type, m=*met1*, t=*mthfd1-1*, c3=*cmt3*, c2=*cmt2*, d=*drm1,2*. Box plot (herein and after): horizontal line, median; edges of boxes, 25th (bottom) and 75th (top) percentiles; error bars, minimum and maximum points within 1.5 × interquartile range. (**g**) Heat map of DNA methylation levels in *mthfd1-1* hypo-DMRs (rows) clustered by methylation levels. (**h**) Overlap of *met1* or *mthfd1-1* CG hypo-DMRs with PCGs or TEs. (**i**) Fluorescence micrographs of representative nuclei from WT and *mthfd1-1*. DNA was stained with DAPI and H3K9me2 was immunostained using Alexa Fluor 647 as secondary antibody. Scale bar, 5 μm. (**j**) Number of nuclei classified by DAPI staining and H3K9me2 immunofluorescence.

To define the effect of the *mthfd1-1* mutation on the different DNA methylation pathways, we calculated differentially methylated regions (DMRs) that had decreased DNA methylation in *mthfd1-1* or the DNMT mutants compared with the WT reference (hypo-DMRs). The comparison of hypo-DMRs clearly showed that CMT3-dependent CHG methylation was the most affected, followed by CMT2- and DRM1,2-dependent CHH methylation (DRM1 is a lowly expressed paralog of DRM2 (ref. 16)), and finally MET1-dependent CG methylation that was the least affected in *mthfd1-1* (Fig. 3a–c). It is noteworthy that *mthfd1-1* DNA methylation levels were also decreased in regions that were only defined as DMRs in the DNMT mutants, but not in *mthfd1-1* (Fig. 3d–f). Moreover, all subsets of *mthfd1-1* hypo-DMRs showed residual DNA methylation, which supports a uniform genome-wide decrease in *mthfd1-1* (Fig. 3d–f). Heat maps of hierarchically clustered CHG and CHH hypo-DMRs further illustrated that DNA methylation levels in *mthfd1-1* are evenly decreased at moderate degrees and thus generally proportional to WT levels (Fig. 3g). The equal ratio of TE- versus PCG-overlapping CG hypo-DMRs in *met1* and *mthfd1-1* is also in agreement with a uniform decrease in genomic DNA methylation in *mthfd1-1* (Fig. 3h). Accordingly, the small subset of *met1* CG hypo-DMRs shared by *mthfd1-1* does not seem to represent site-specific MTHFD1 function, but is more likely due to stringent criteria for DMR calling, which were not met by the majority of sites in *mthfd1-1* despite reduced CG methylation levels (Fig. 3a,d). In the CHH context, RdDM and the CMT2 pathway were both affected by *mthfd1-1* (Fig. 3c,g). The chromosomal distribution of *mthfd1-1* CHH hypo-DMRs, with high densities inside and at the peripheries of the pericentromeric regions, and lower densities in the chromosomal arms, reflects the overlaps with the alternative CHH methylation pathways (Supplementary Fig. 5d). In summary, the DNA methylation analysis of *mthfd1-1* revealed a rather uniform genome-wide decrease, which is most pronounced in the CHG and CHH context.

DNA methylation by CMT3, CMT2 and RdDM is functionally linked to histone methylation, because CMT2 and CMT3 directly bind H3K9me in a feedback loop with the histone methyltransferases KYP/SUVH4, SUVH5 and SUVH6 (refs 5, 6), and Pol IV is recruited to chromatin by K3K9me-binding SHH1 (ref. 10). To test if H3K9me is also affected in *mthfd1-1*, we analysed H3K9 dimethylation (H3K9me2) by immunofluorescence. Nuclei of *mthfd1-1* mutants showed a strong decrease in H3K9me2, but the majority of the nuclei still contained DNA-dense chromocenters visualized by DAPI staining (Fig. 3i,j). Therefore, the strong decrease in CHG and CHH methylation in contrast to the small decrease in CG methylation is likely explained by the combined effect of impaired DNA and H3K9 methylation.

**Figure 4:**
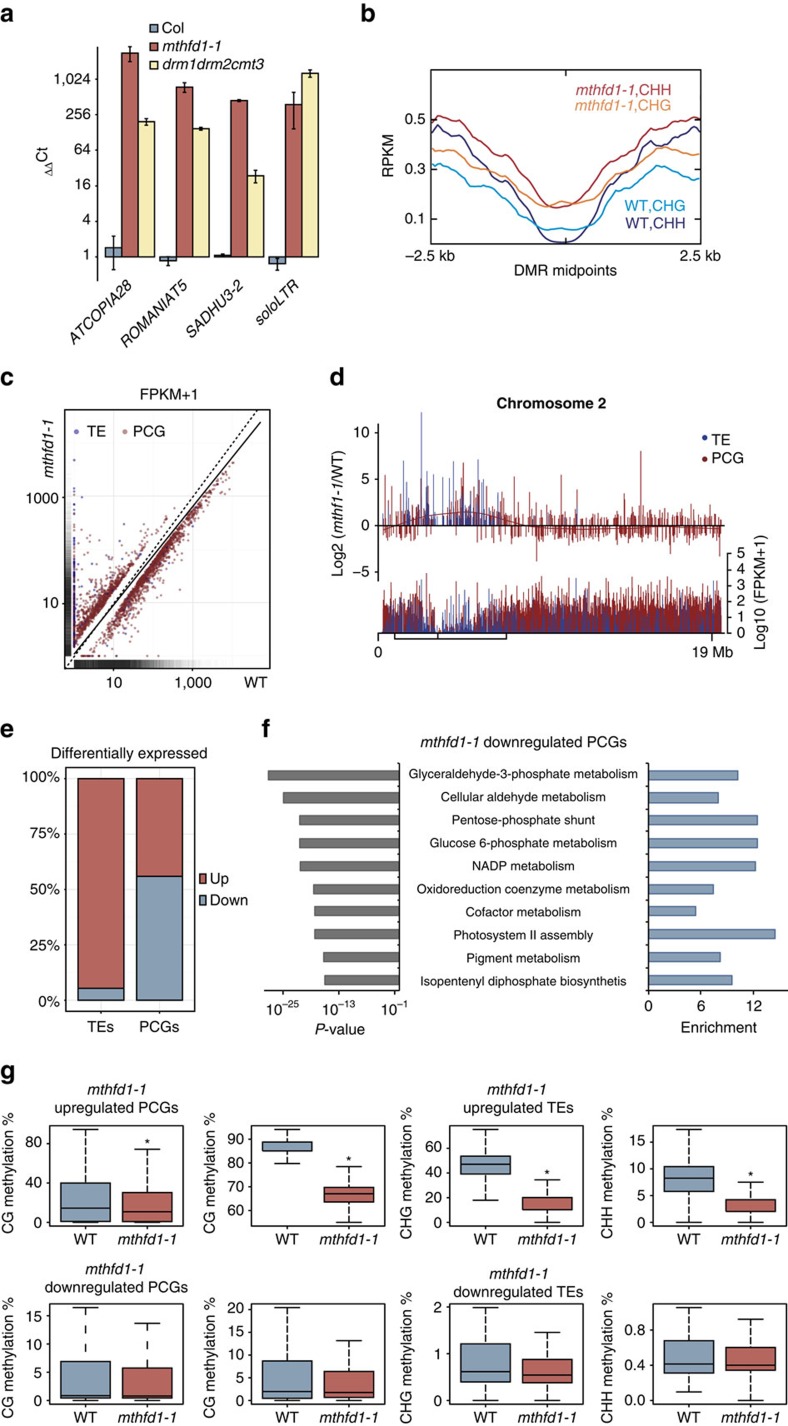
*mthfd1-1* mutants show loss of TE silencing and pleiotropic transcriptional deregulation of PCGs. (**a**) Quantitative RT-PCR analysis of four exemplary TEs. Transcript levels are normalized to *ACTIN7* and relative to WT, and mean values±s.e.m. (*n*=3) are shown. (**b**) Average distribution of normalized RNA-seq reads (RPKM) over *mthfd1-1* hypo-DMRs in CHG or CHH context. *X* axis indicates distance from the DMR midpoints. (**c**) Scatter plot showing normalized transcript levels (FPKM+1) of differentially expressed PCGs and TEs in *mthfd1-1* versus WT. Solid line: linear regression through all TEs and PCGs. Dashed: identity line. Marginal density plots: distribution of all TEs and PCGs in WT (*x* axis) and *mthfd1-1* (y-axis). (**d**) Distribution of average normalized transcript levels (FPKM+1) of all TEs and PCGs from *mthfd1-1* and WT (lower panel) and fold change in normalized transcript levels of differentially expressed PCGs and TEs (upper panel) along chromosome 2. Box and vertical line inside box mark pericentromeric region and centromere, respectively. (**e**) Fraction of TEs and PCGs significantly up- or downregulated in *mthfd1-1*. (**f**) Enrichment score and statistical significance of GO processes annotated to *mthfd1-1*-downregulated PCGs. (**g**) Methylation levels over *mthfd1-1* differentially expressed PCGs and TEs in different sequence contexts.

To test if loss of DNA methylation in *mthfd1-1* led to transcriptional derepression, we analysed the expression levels of different retrotransposons that were previously identified as upregulated in *drm1,2 cmt3* triple mutants^13^. Quantitative reverse transcription (RT)-PCR showed that these TEs are also strongly induced in *mthfd1-1* (Fig. 4a and Supplementary Data 1). Transcriptome analysis of *mthfd1-1* and WT by RNA-seq confirmed that average transcript levels over CHH and CHG hypo-DMRs are higher in *mthfd1-1* than in WT (Fig. 4b). Many transcripts from TEs that were silenced in WT were highly abundant in *mthfd1-1*, whereas transcriptional differences of PCGs were more even and showed a slight tendency towards higher transcript levels in WT (Fig. 4c–e). Correspondingly, pericentromeric regions showed many differentially upregulated TEs and—to a lesser degree—PCGs in *mthfd1-1*, whereas chromosome arms contained approximately equal distributions of up- and downregulated PCGs (Fig. 4d,e). The differentially upregulated TEs in *mthfd1-1* belonged to class I, as well as class II transposons. Among the differentially upregulated TEs, members of the LTR/Gypsy family were overrepresented, and members of the RC/Helitron family were under-represented compared with the genomic distribution (Supplementary Fig. 6). In summary, the transcriptional analyses have shown that the loss of DNA methylation in *mthfd1-1* led to derepression of transposons and genes (Fig. 4g), predominantly in the pericentromeric region (Fig. 4d). The observed transcriptional changes in the chromosome arms seem to be mainly a consequence of pleiotropic effects of impaired MTHFD1 function. This is supported by an analysis of GO terms annotated to genes that are significantly downregulated in *mthfd1-1* compared with WT (Fig. 4f and Supplementary Data 2). The 10 statistically most significantly enriched biological processes indicate that MTHFD1 serves important functions in sugar metabolism, isoprenoid synthesis, redox homoeostasis and photosynthesis. Since *mthfd1-1*-downregulated genes did not show a significant loss of DNA methylation, the overall effects on transcript abundance caused by decreased gene body methylation in *mthfd1-1* are likely to be negligible (Fig. 4g).

### *mthfd1-1* mutants show accumulation of S-adenosylhomocysteine

**Figure 5:**
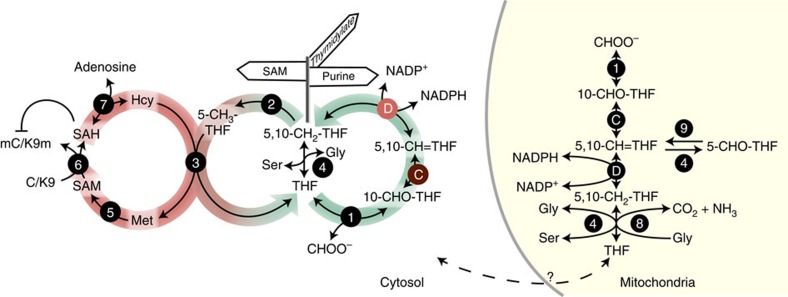
Schematic representation of plant SAM and folate metabolism in the cytosol and mitochondria. One-carbon enters the cytoplasmic folate cycle (green) either through formyltetrahyrofolate synthetase (1) or SHMT (4); MTHFD1 reversibly interconverts 10-CHO-THF to 5,10-CH2-THF by cyclohydrolase (C) and NADP^+^-dependent dehydrogenase (D) activity. 5,10-CH2-THF serves for thymidylate synthesis or is converted by methylenetetrahyrofolate reductase (2) to 5-CH3-THF, which enters the Met cycle (red) and serves for Hcy remethylation to Met by methionine synthase (3) (ref. 17). SAM synthetase (5) converts Met to SAM, which is further converted to SAH (6) during methylation of cytosines, H3K9 and so on. SAH is a competitive inhibitor of methyltransferases (6) and is recycled to Hcy by SAH hydrolase (7) (ref. 23). In mitochondria, one-carbon is transferred to THF during the oxidation of Gly by the glycine decarboxylase complex (8), but surplus of Gly due to photorespiration leads to consumption of one-carbon by SHMT during serine production^30^. 5-CHO-THF, a byproduct of SHMT, is metabolized by mitochondrial 5-formyltetrahyrofolate cycloligase in order to re-enter the folate cycle (9) (ref. 59). Shuttle of THF between mitochondria and the cytosol has been described in other organisms, but remains uncharacterized in plants.

**Figure 6:**
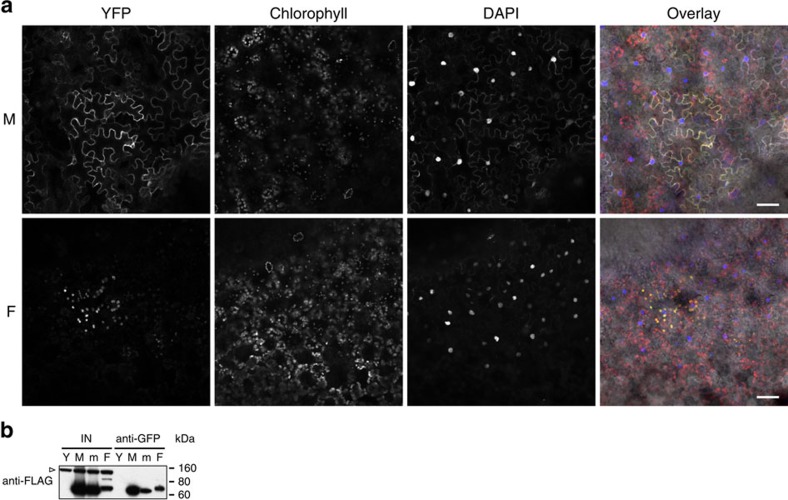
Cytoplasmic localization of MTHFD1-YPET-3xFLAG. (**a**) Confocal micrographs of MTHFD1-YPET-3xFLAG (M) and FOLD4-YPET-3xFLAG (F) transiently expressed in *N. benthamiana*. Excitation (*λ*, nm)/filters (*λ*, nm): YFP=514/519–559, chlorophyll=488/630–730, DAPI=405/409–530, and fluorescence overlay with bright field. Scale bars, 50 μm. (**b**) Western blot using anti-FLAG antibody against anti-GFP-immunopurified extracts from *N*. *benthamiana* (IN) transiently expressing free YFP (Y), MTHFD1-YPET-3xFLAG (M), MTHFD1_R175Q-YPET-3xFLAG (m), or FOLD4-YPET-3xFLAG (F). Arrowhead indicates unspecific binding of anti-FLAG (shown as loading reference).

**Figure 7:**
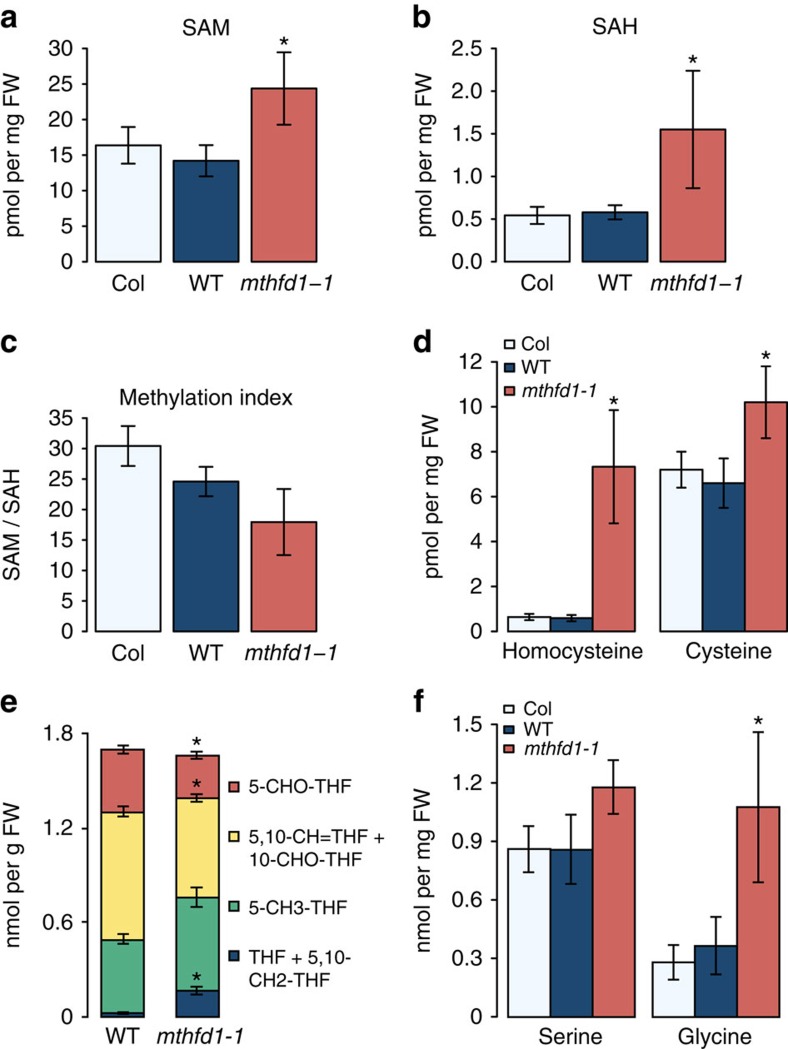
*mthfd1-1* mutants show impaired one-carbon cycle. (**a**–**f**) Steady-state levels of SAM (**a**), SAH (**b**), Methylation Index (MI) (**c**), selected thiols (**d**), folates (**e**) and selected amino acids (**f**) in leaves of Col, WT and the *mthfd1-1* mutant. Data represent means±SD. Asterisks indicate significant differences determined by Student's *t*-test (*P*<0.05, *n*≥3).

*Arabidopsis* MTHFD1 contains two highly conserved protein domains, a catalytic domain in the N-terminal half, and a NAD(P^+^)-binding domain of the Rossmann fold superfamily in the C-terminal half (Fig. 1b). Therefore MTHFD1 is probably required for the interconversion of tetrahydrofolate (THF) species in one-carbon metabolism of *Arabidopsis*. Members of the bifunctional enzyme family catalyse the reversible interconversion of 5,10-methylenetetrahydrofolate (5,10-CH2-THF) to 5,10-methenyltetrahydrofolate (5,10-CH=THF) (NADP^+^-dependent dehydrogenase activity) and further to 10-formyltetrahydrofolate (10-CHO-THF) (cyclohydrolase activity) (Fig. 5) (ref. 17). These enzymatic activities have previously been detected in plant extracts^18^. The *Arabidopsis* genome encodes four homologues, the mitochondrial MTHFD2/FOLD1 (AT2G38660), FOLD3 (At4g00600) and FOLD4 (At4g00620), which are putatively plastidic, and MTHFD1/FOLD2, which lacks an N-terminal targeting peptide and is presumably localized in the cytoplasm (Supplementary Fig. 7) (refs 19, 20, 21). We confirmed the subcellular localization of the latter two homologues by expression and *in vivo* imaging of the full-length fusion proteins MTHFD1-YPET-3xFLAG, MTHFD1_R175Q-YPET-3xFLAG and FOLD4-YPET-3xFLAG in *Nicotiana benthamiana* (Fig. 6a,b and Supplementary Fig. 8), showing that MTHFD1 is predominantly in the cytoplasm. The Met cycle is exclusively localized in the cytoplasm and is required for the synthesis of SAM, which serves as methyl donor for many transmethylation reactions, including those catalysed by histone and DNMTs (Fig. 5). During transmethylation SAM is converted to SAH, which is further processed into adenosine and Hcy by the SAH hydrolase (SAHH) (Fig. 5). Hcy is recycled to Met by 5-CH3-THF-dependent transmethylation activity of methionine synthase and can serve for a new round of SAM synthesis (Fig. 5) (refs 14, 22). To test if the DNA methylation defects in *mthfd1-1* are caused by changes in the Met cycle, we analysed the levels of SAM, SAH and Hcy, as well as cysteine in *mthfd1-1*, WT and Col leaves. SAM and SAH were both significantly increased in *mthfd1-1*, but the stronger increase in SAH levels led to an overall decrease of the methylation index (MI=SAM/SAH) (Fig. 7a–c). MI is an important measure of the organismal methylation status, because SAH is a strong competitive inhibitor of SAM-dependent transmethylation^23^. Because of the low intracellular concentration and high affinity for methyltransferases, it has been suggested that even small changes in the MI can lead to a reduction in transmethylation activity^24^. Therefore, it is likely that the decreased MI in *mthfd1-1* leads to decreased activities of DNA and histone methyltransferases, as reflected by the observed DNA and histone methylation defects. This is further supported by a 12-fold increase in Hcy (and an associated increase in cysteine levels) in *mthfd1-1* (Fig. 7d), because Hcy accumulation leads to inhibition of SAH hydrolysis and consequently to a lower MI^22^,^25^.

### Regulation of folate homoeostasis by MTHFD1

Analysis of folate metabolites in leaves of *mthfd1-1* and WT showed that total folate content did not differ significantly, but *mthfd1-1* had an 8.8-fold increase in THF+5,10-CH2-THF (these two compounds cannot be distinguished by the analysis). On the other hand, the levels of 5,10-CH=THF, which also include the 10-CHO-THF pool, were reduced by 30% and 5-CHO-THF levels were also ∼33% lower (Fig. 7e). Unexpectedly, there was no significant difference in 5-CH3-THF contents, which is the product of 5,10-CH2-THF reductase that serves as co-substrate for the methylation of Hcy to Met and constitutes the most abundant active THF species^26^. The low levels of the oxidized folates, 10-CHO-THF+5,10-CH=THF and 5-CHO-THF, suggest an impairment in the dehydrogenase and cyclohydrolase activities towards the formation of 10-CHO-THF. On the basis of the crystal structure and site-directed mutagenesis of human MTHFD1, the conserved arginine that is mutated in *mthfd1-1* (R175Q), forms a hydrogen bond with NADP^+^ and is required for dehydrogenase, but not cyclohydrolase activity^27^,^28^. Therefore, we anticipate that the R175Q mutation has a similar effect in *mthfd1-1* mutants. Accordingly, the accumulation of the reduced forms THF+5,10-CH2-THF in *mthfd1-1* might reflect reduced conversion of 5,10-CH2-THF to 5,10-CH=THF, which is in agreement with the homology-based prediction that the R175Q mutation affects NADP^+^ binding and dehydrogenase activity^27^,^28^. The decreased pool of oxidized THFs further suggests that one-carbon supply from formate via FTHFS and reverse cyclohydrolase by MTHFD1 is not sufficient to compensate for reduced MTHFD1 dehydrogenase activity (Fig. 5). This is in accordance with metabolic analyses showing relatively low one-carbon flow from formate to serine (Ser)^29^.

Ser can serve as a one-carbon source through the reversible enzymatic activity of serine hydroxymethyltransferase (SHMT), which converts Ser and THF to 5,10-CH2-THF and glycine (Gly) (Fig. 5). In addition, the Gly decarboxylase complex converts Gly and THF to 5,10-CH2-THF, carbon dioxide and ammonia during photorespiration, which in turn can lead to Ser synthesis by SHMT in the mitochondria^30^ (Fig. 5). We analysed amino-acid levels in rosette leaves and found a threefold increase of Gly levels in *mthfd1-1* compared with WT and Col controls, whereas Ser levels were only slightly increased in *mthfd1-1* (Fig. 7f). In accordance with our folate analysis, the lower Ser/Gly ratio in *mthfd1-1* might be due to increased SHMT activity towards 5,10-CH2-THF formation^19^. Furthermore, *mthfd1-1* mutants showed a 5.4-fold and 1.9-fold increase in proline and Met levels, respectively (Supplementary Fig. 9). The increase in Met might be due to increased *de novo* synthesis (which occurs exclusively in plastids) in response to impaired Met recycling, whereas proline accumulation is typically observed as a stress response^31^.

**Figure 8:**
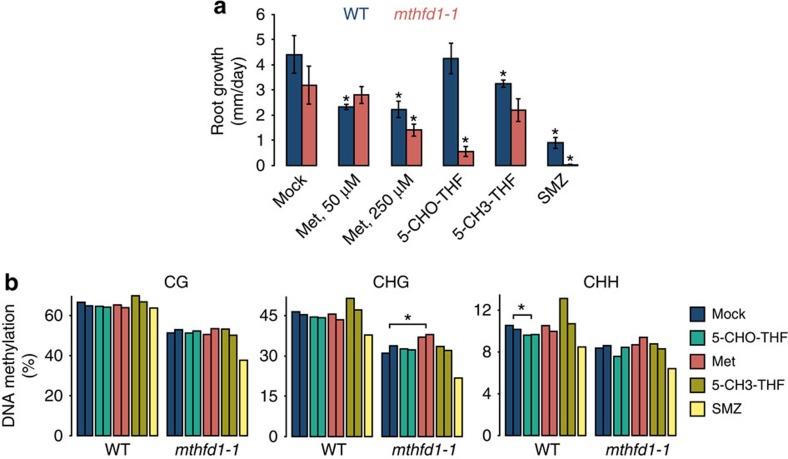
*mthfd1-1* mutants are hypersensitive to exogenous 5-CHO-THF but tolerant to exogenous methionine. (**a**) Root growth of seedlings on solid media containing mock, Met, 0.5 mM 5-CHO-THF, 0.5 mM 5-CH3-THF or 5 μM SMZ. Mean values±s.d. (*n*=3) are shown. (**b**) Average CG, CHG and CHH methylation levels at previously defined *mthfd1-1* CG, CHG and CHH hypo-DMRs, respectively, in seedlings grown for 14 days on solid media containing mock, 0.1 mM Met, 0.5 mM 5-CHO-THF, 0.5 mM 5-CH3-THF or 5 μM SMZ. Two biological replicates are shown, except for SMZ. * indicates significant difference between mock and chemical treatment (*P*<0.05, Student's *t*-test).

We sought to manipulate one-carbon metabolism in *mthfd1-1* by exogenous application of metabolites involved in the folate and Met cycle and monitor root growth and DNA methylation effects. Previously it has been shown that growth phenotypes, DNA methylation and epigenetic silencing defects caused by decreased activated THF pools because of impaired plastidic folylpolyglutamate synthetase (FPGS1), or chemical inhibition of folate synthesis by sulfamethazine (SMZ) were complemented by exogenous application of 5-CHO-THF or Met^32^,^33^,^34^,^35^. Application of 5-CHO-THF, as well as 5-CH3-THF, tests for defects in *mthfd1-1* caused by reduced folate availability before the flow of one-carbon into the Met cycle, whereas application of Met tests for defects in the Met cycle (Fig. 5), and SMZ was used as a control. In contrast to previously described complementation of *fpgs1* mutants, 5-CHO-THF strongly inhibited root growth of *mthfd1-1* seedlings without showing an inhibitory effect on WT seedlings (Fig. 8a). Although analysis of global DNA methylation levels revealed that these are largely independent of the observed root growth responses, average *mthfd1-1* CHG methylation over the previously defined CHG hypo-DMRs significantly increased upon Met application. On the other hand, 5-CHO-THF, as well as 5-CH3-THF, did not rescue the DNA methylation defects in *mthf1-1* (Fig. 8b). These results indicate that, in contrast to the *fpgs1* mutants and SMZ inhibition^34^,^35^, the DNA methylation defects in *mthfd1-1* are probably not the mere result of diminished folate pools, but rather point towards an inhibition of methionine synthase.

## Discussion

Methylation patterns in the *Arabidopsis* genome are remarkably stable not only from one generation to the next but also at evolutionary timescales^36^,^37^,^38^,^39^. Comparative genomics and genome-wide association studies have linked DNA methylation and phenotypic variation in Brassicaceae to genetic polymorphisms in the DNA methylation machinery^40^,^41^, and consequently support an adaptive role of spontaneous epigenetic changes. For example, two independent studies have revealed that different alleles of *CMT2* and the concomitant differences in CHH methylation are associated with climate adaptation^40^,^42^. Here we have identified an EMS-induced polymorphism in the essential folate metabolic enzyme MTHFD1 from *Arabidopsis*, which causes a strong, genome-wide decrease in DNA methylation. This finding highlights that DNA methylation patterns in *Arabidopsis* not only depend on the pathways and catalytic activities of the DNMTs but also on the metabolic network that regulates the availability of the methyl donor SAM and the adequate functioning of the activated methyl cycle. It is therefore conceivable that regulatory mechanisms have evolved, which connect nutritional changes to epigenetic gene regulation by DNA and histone methylation. Although direct examples in plants are still lacking, it has been shown that a folate-rich diet in mice leads to changes in coat colour of the offspring that is caused by altered expression of the *agouti* gene due to increased DNA methylation of a transposon in the *agouti* locus (*A*^*vy*^)^43^. This finding illustrates an example of how such regulatory mechanisms could work.

The EMS-allele *mthfd1-1* was identified through a genetic screen for mutants that simultaneously affect CHG and CHH methylation. Correspondingly, our genome-wide BS-seq analysis of *mthfd1-1* mutants revealed extensive hypomethylation in CHG and CHH sequence contexts. In contrast, loss of CG methylation was comparatively low. We therefore reason that the feedback regulation between CHG/CHH and H3K9 methylation is particularly prone to changes in one-carbon metabolism, because transmethylation by DNA and histone methyltransferases are both SAM-dependent. Accordingly, *mthfd1-1*, as well as previous analyses of *fpgs1* mutants and plants treated with SMZ^34^,^35^, showed reduced H3K9me2. Because of the mechanistic interdependence of non-CG and H3K9 methylation^5^, it is difficult to tell whether histone or DNA methylation is more directly affected by impaired one-carbon metabolism. The predominant effect on CHG methylation might suggest that loss of H3K9me2 is the primary defect. However, the fact that CG methylation is also decreased in *mthfd1-1,* a type of methylation that is not linked with H3K9 methylation, suggests a general inhibition of the enzymatic activity of different methyltransferases, including MET1.

Our transcriptome analysis demonstrated that loss of DNA methylation in *mthfd1-1* mutants leads to derepression of TEs and a generally higher abundance of transcripts from the pericentromeric heterochromatin compared with WT. With respect to the almost equal numbers of up- and downregulated PCGs, loss of gene body methylation can only account for some of the observed changes, whereas the majority of differential gene expression is probably caused by pleiotropic effects of impaired MTHFD1 function. Secondary to its role in one-carbon metabolism, a shortfall of NADP^+^ conversion by MTHFD1 is expected to severely disturb the redox state^44^. Accordingly, GO term analysis revealed a significant enrichment of genes involved in cell redox homoeostasis, oxidative pentose phosphate pathway and glycolysis (Fig. 4f and Supplementary Data 2) (ref. 45).

MTHFD1 related proteins in different species have mono-, bi- or trifunctional enzymatic activity. Yeast and mammalian cytosolic homologues, known as C1-THF synthases, are trifunctional and reversibly catalyse the stepwise oxidation from 5,10-CH2-THF to 10-CHO-THF, which serve for thymidylate/pantothenate and *de novo* purine/*N*-formylmethionine synthesis, respectively, and the conversion of 10-CHO-THF to THF and formate (reverse FTHFS activity) (Fig. 5) (refs 14, 46, 47). Bifunctional forms, which are found in certain bacteria and in plants, lack the FTHFS activity^18^,^48^,^49^. Moreover, methylenetetrahydrofolate reductase converts 5,10-CH2-THF to 5-CH3-THF and thereby directs activated methyl towards SAM (Fig. 5). As such, the reversible enzymatic activity of MTHFD1 channels one-carbon into different pathways and acts as a crucial regulatory hub (Fig. 5). Correspondingly, functional mutations, such as in *mthfd1-2*, have severe pleiotropic effects and are mostly lethal. This is in contrast to the subtle morphological defects generally observed in epigenetic *Arabidopsis* mutants, for example, *drm1 drm2 cmt3* triple knockout mutants^50^, and denotes that inhibition of MTHFD1 leads to pleiotropic morphological defects that are independent of its impact on DNA and histone methylation. The essential nature of MTHFD1 further indicates that the additional three *Arabidopsis MTHFD* homologues have plastid- and mitochondrion-specific functions that cannot compensate for a loss of cytoplasmic MTHFD1 function.

Because of its role in nucleotide biosynthesis and DNA methylation, folate metabolism is of central relevance in cancer research, as exemplified by the therapeutical use of antifolates^51^. Polymorphisms in human MTHD1 C1-THF synthase have been associated with cancers, as well as neural tube defects and other illnesses^51^. Interestingly, one polymorphism (R173C) resides in the same conserved residue that is mutated in the EMS-allele *mthfd1-1* and was linked to severe combined immunodeficiency, megaloblastic anaemia and altered Met metabolism, including Hcy accumulation^52^. Analyses of fibroblasts harbouring this mutation showed signs of DNA damage and uracil misincorporation into DNA due to impaired *de novo* thymidylate synthesis^52^. Interestingly, the mutation had the strongest impact on one-carbon flow towards the Met cycle^52^. Impaired dehydrogenase activity was partially compensated by increased SHMT activity, as well as increased salvage thymidylate synthesis, whereas *de novo* purine synthesis was not affected^52^. The study did not include DNA methylation analyses, but given the conserved function of MTHFD1 and shared effects on Hcy remethylation, our results predict that DNA methylation is affected by the MTHFD1 R173C mutation and might be involved in certain types of severe combined immunodeficiency and megaloblastic anaemia. In reverse, analogous redirection of one-carbon flow towards nucleotide synthesis at the expense of Hcy remethylation is a possible explanation of the defects observed in *mthfd1-1* and was also suggested to occur upon methotrexate-induced THF depletion in *Arabidopsis*, based on folate measurements and transcriptional analyses^53^.

Hcy accumulation and decreased MI, as observed in *mthfd1-1* and previous studies^32^,^33^,^34^,^35^, are hallmarks of impaired Hcy remethylation due to impaired folate metabolism. Increased Hcy levels lead to decreased SAH hydrolase activity and accumulation of SAH, which competitively inhibits SAM-dependent transmethylation, including DNA and histone methylation^54^,^55^.

Decreased flux of one-carbon towards the remethylation of Hcy should intuitively lead to decreased levels of Met, yet we observed increased cellular Met in *mthfd1-1*. However, primary metabolites, in particular the sulfur amino acids Cys and Met, are often controlled by multiple layers of regulatory circuits, as exemplified by the *sir1-1* mutant, which also show an increased Met steady level despite a 20-fold decreased flux of sulfur through the assimilatory sulfate reduction pathway^56^. This increase was the result of decreased flux into Met sinks, due to an attenuation of translation and growth in *sir1-1* (ref. 56). To that effect, it is conceivable that Met levels were increased in the *mthfd1* mutant because histone and DNA methylation (two major one-carbon sinks) and growth were decreased. Moreover, increased Met levels might also be due to increased Met *de novo* synthesis. Accordingly, the transcriptome analysis of *mthfd1-1* shows a 4.3-fold increase in transcripts corresponding to *MRU1* (At5g35490), which is also upregulated in the Met over-accumulating mutant *mto1-1* (ref. 57).

In the cases of chemically inhibited THF synthesis or impaired THF polyglutamylation, exogenous application of 5-CHO-THF, which is readily assimilated and metabolized to active THF forms in *Arabidopsis*^53^,^58^, successfully reversed the feedback inhibition of transmethylation and TGS^34^,^35^. Interestingly, 5-CHO-THF feeding to *mthfd1-1* mutants did not complement the DNA methylation defect and had a strong adverse effect on root growth. This hypersensitivity could be attributable to an inhibitory effect of 5-CHO-THF on SHMT^59^. As in R173C fibroblasts^52^, it is likely that the supply of 5,10-CH2-THF in *mthfd1-1* mutants depends on cytosolic SHMT. Although cell compartmentalization demands a cautious interpretation of the metabolic profiles, the observed decrease in 5-CHO-THF levels by 33% in *mthfd1-1* versus WT might have led to an increase in SHMT activity. Since the reaction equilibrium catalysed by SHMT favours Gly production^60^, increased SHMT activity might have contributed to the threefold increase in steady-state Gly levels observed in *mthfd1-1* versus WT. An inhibition of SHMT by exogenously applied 5-CHO-THF would accordingly cut off the cytosolic 5,10-CH2-THF supply and explain the enhanced root growth defect in *mthfd1-1* mutants. It is noteworthy that we did not observe an enhanced DNA methylation defect upon 5-CHO-THF feeding, which suggests that even under normal growth conditions SHMT-dependent 5,10-CH2-THF production is unable to perpetuate the Met cycle in *mthfd1-1*. This is further supported by the lack of phenotypic rescue of *mthfd1-1* by exogenous 5-CH3-THF. On the other hand, exogenous Met partially restored global CHG methylation, which together with the folate quantifications and feeding experiments suggests that transmethylation in *mthfd1-1* is impaired due to an inhibition of Hcy remethylation, as opposed to limited availability of folate intermediates^34^,^35^ or inhibition of SAHH^54^. As such, the described DNA methylation and gene regulatory defects in *mthfd1-1* highlight a central regulatory role of MTHFD1 in one-carbon distribution towards different cell physiological processes.

## Methods

### Plant material

All plants used in this study were of the Columbia-0 ecotype. T-DNA insertion mutants *mthfd1-2* (WiscDsLox244C04), *mthfd1-3* (SALK_015165) and *mthfd1-4* (SALK_039538) were obtained from the *Arabidopsis* Biological Research Center (Ohio State University). Genotypes were analysed by PCR using primers listed in Supplementary Table 2. The triple mutant *drm1 drm2 cmt3* and the WT transgenic line carrying the *SDC*_*pro*_*-GFP* fusion construct were published previously^4^,^13^. *mthfd1-1* mutants have been backcrossed with WT plants carrying *SDC*_*pro*_*-GFP*. Plants were grown in the greenhouse at long day light cycles, unless stated differently.

### Genetics screening and mapping analyses

WT seeds (2,000) were suspended in 0.3% EMS solution for 13 h with rotation, washed with water and planted on soil. Approximately 1,000 independent M2 populations were collected and screened for GFP fluorescence using a Leica MZ16F Fluorescence Stereomicroscope equipped with a GPF Plus filter. Pictures were taken with a DFC300 FX digital camera. For mapping and identification of EMS mutations, mutant #162 was crossed with WT L*er* and 10-days-old F2 seedlings grown on media containing 1 × Murashige and Skoog basal salt mixture (MP) and 20 μg ml^−1^ glufosinate ammonium (Sigma) were analysed for GFP expression. Genomic DNA was isolated from pooled tissue of 50 GFP-positive F2 mutants and analysed by whole-genome re-sequencing for co-segregating single-nucleotide polymorphisms between Col and *L*er^13^. Primer sequences of CAPS markers for co-segregation analyses are shown in Supplementary Table 2.

### Local DNA methylation analyses

Genomic DNA was isolated from aerial tissue of 4–5-weeks-old plants. The *MEA-ISR* probe for DNA blot analysis was amplified using primers JP980 and JP981 (Supplementary Table 2) (ref. 15). Vertically uncropped images of all blots and gels shown in this study are provided in Supplementary Fig. 10. Chop-PCR analysis of *AtSN1* was performed by real-time PCR using primers JP6349 and JP6350 (Supplementary Table 2) (ref. 61). For DNA methylation analysis of the transgenic *SDC* promoter, DNA was BS converted using EZ DNA Methylation Gold kit (Zymo Research) and PCR amplified using primers listed in Supplementary Table 2. PCR fragments were cloned into pCR2.1-TOPO (Thermo Fisher Scientific), and 20 clones per genotype were sequenced.

### Whole-genome bisulfite sequencing

Genomic DNA was extracted from rosette leaves of 3-weeks-old plants using DNeasy Plant Mini Kit (Qiagen) and fragmented into 200 bp average size with a Covaris S2 sonicator. Next, fragmented DNA was end repaired, adenylated and ligated with TruSeq DNA LT adapters (Illumina) using NEBNext DNA library prep reagent set (NEB). Subsequently, BS conversion was performed with CpGenome DNA modification kit (Millipore). Libraries were amplified using PCR primer cocktail (Illumina) and Pfu Turbo Cx hotstart DNA polymerase (Agilent). Sequencing was performed on a HiSeq 2000 platform at 50 bp length. Identical reads were removed and unique reads were aligned to the *Arabidopsis* reference genome (TAIR10) using BSMAP 2.87 (ref. 62). Read statistics are listed in Supplementary Table 3. Data for mutants other than the *mthfd1-1* were obtained from GSE39901 (ref. 63). Methylation levels were calculated as #C/(#C+#T). DMRs were defined by dividing the genome into 100 bp bins and comparing mutants and WT by the number of methylated and unmethylated Cs with at least four Cs covered using Fisher's exact test and cutoffs of Benjamini–Hochberg corrected false discovery rate<0.01. Moreover, absolute methylation difference of each bin had to be at least 0.4, 0.2 and 0.1 for CG, CHG and CHH, respectively. Heat maps of DMRs were generated by ‘pheatmap' package in R software and clusters were grouped by the complete linkage method with Euclidean distance measurement. Venn diagrams were generated by calculating overlaps of 100 bp DMRs.

### RNA analyses

Total RNA was isolated with TRIzol (Thermo Fisher Scientific) from 0.1 g of rosette leaves from 3-weeks-old plants. For real-time RT-PCR analysis, 2 μg of DNase I-treated total RNA were reverse-transcribed with SuperScript III (Thermo Fisher Scientific) and cDNA was amplified at target loci (primers listed in Supplementary Table 2) using iQ SYBR Green Supermix (Bio-Rad) and a Mx3005P qPCR system (Agilent Technologies).

For RNA-seq analysis, unstranded libraries from poly-A-tailed RNA were generated according to the manufacturer's instructions (Illumina TruSeq) and sequenced with the HiSeq 2,000 platform at 50 bp length. Reads were mapped to the TAIR10 genome with TopHat2 (ref. 64) using defaults settings, except that intron length was set to 40–5,000. Read statistics are listed in Supplementary Table 3. Fragments per kilobase of exon per million fragments mapped (FPKM) values and differential gene expression were analysed with Cufflinks^65^ using default settings, except that maximum intron length was set to 5,000 and the –u option was used. The reference annotation for Cufflinks analysis was downloaded from TAIR and combined genes, including pseudogenes and TE genes, and TEs. GO term enrichment in genes that were significantly down regulated in *mthfd1-1* compared with WT by at least twofold was analysed with GOrilla^66^, using all *Arabidopsis* PCGs as background list.

### Immunofluorescence analysis

Nuclei from rosette leaves of 3-weeks-old plants were immunostained with anti-H3K9me2 primary (Abcam ab1220, 5 μg ml^−1^) and Alexa Fluor 647-conjugated anti-mouse IgG secondary (Thermo Fisher Scientific A-31571, 10 μg ml^−1^) antibodies, and counterstained with DAPI (1 μg ml^−1^) (ref. 13). Stained nuclei were imaged with a LSM 710 confocal microscope (Zeiss), with a C-Apochromat × 40/1.2 W Corr M27 objective and detection at *λ* (nm)=410–504 (DAPI) and *λ* (nm)=653–680 (Alexa Fluor 647).

### Subcellular localization

To generate C-terminally tagged translational fusion proteins MTHFD1-YPET-3xFLAG, MTHFD1_R175Q-YPET-3xFLAG and FOLD4-YPET-3xFLAG, genomic DNA from Col and *mthfd1-1* was amplified with primer pairs JP14184/5 and JP14190/1 (Supplementary Table 2), spanning the entire ORF (excluding Stop) and 1147 and 866 bp 5′ of the ORF of *MTHFD1* and *FOLD4*, respectively. The amplified products were digested with XhoI & SpeI or SalI & SpeI and ligated with the plasmid pBJ36 (ref. 67), which has been linearized with XhoI & XbaI and contained an insertion of *YPET-3xFLAG* on the 3′-side of the XbaI site. Not1 fragments from the resulting plasmids were inserted into the Not1 site of the binary vector pMLBART^67^. Overnight cultures of transformed *Agrobacterium tumefaciens* strain ASE were adjusted to OD600=0.3 and coinfiltrated with p19 into *N. benthamiana* leaves^68^. Leave discs were imaged 4 days after infiltration with a LSM 710 confocal microscope (Zeiss), using a Plan-Apochromat × 20/0.8 M27 objective and sequential scanning at excitation/detection *λ* (nm)=514/519–559 (YFP), 488/630–730 (chlorophyll) and 405/409–530 (DAPI).

### Immunopurification and western blot analysis

Agro-infiltrated *N. benthamiana* leaves (0.5 g) were ground in liquid nitrogen, and ground tissue was resuspended in 3 ml of IP buffer (50 mM Tris pH7.6, 150 mM NaCl, 5 mM MgCl2, 5% (vol/vol) glycerol, 1% Tergitol (Type NP-40, Sigma), 2.8 mM β-mercaptoethanol, 1 μg ml^−1^ pepstatin, 1 mM PMSF and 1 × protease inhibitor mixture tablet (Roche, 14696200)). Cleared lysates were incubated with 4 μl of anti-GFP antibody (A-11122, Molecular Probes), followed by 50 μl of Dynabeads Protein G (Thermo Fisher Scientific) at 4 °C for 1 h each. Western blotting was performed with anti-FLAG M2-Peroxidase (horseradish peroxidase) antibody (A8592-1MG, Sigma, 1:7,500 dilution).

### Quantification of metabolites

Thiols, amino acids and adenosine nucleotides were extracted with 0.5 ml of 0.1 M hydrochloric acid from 0.1 g of in liquid nitrogen grinded rosette leaves from 4-weeks-old plants (*n*=6) grown at long day light and 21 °C. Amino acids and thiol were labelled with AccQ-Tag (Waters) and monobromobimane (Callbiochem), respectively, and quantified after separation by reverse phase chromatography^69^. SAM and SAH were converted by chloroacetaldehyde treatment to their fluorescent etheno-derivates and quantified according to Burstenbinder *et al.*^70^ after separation on a Gemini-NX C18 column (150 × 3 mm, 5 μm, 110 A, Phenomenex, Germany) connected to a Waters 600 HPLC system with a flow rate of 1 ml min^−1^ using the following gradient: 5 min 100% buffer A (50 mM tri-sodium phosphate deodecahydrate, 10 mM sodium 1-heptane sulfonate, 4% acetonitrile, pH 3.2); linear gradient for 15 min to 15% buffer B (pure acetonitrile); 7 min linear gradient to 90% buffer B; and 3 min 90% buffer B followed by re-equilibration of the column in 100% buffer A for 20 min.

### Folate analysis

*Arabidopsis* rosette leaves (∼0.15 g) were pulverized in a mortar with addition of liquid N_2_ and homogenized with 10 ml folate extraction buffer (50 mM HEPES, 50 mM CHES, 10 mM β-mercaptoethanol, 2% Na-ascorbate (p/v), pH 7.9). The extracts were deglutamylated with a recombinant conjugase from *Arabidopsis* (100 μg AtGGH2 g^−1^ sample) for 1 h at 37 °C. Folates were purified by affinity chromatography using folate-binding columns. Purified folates were separated by liquid chromatography (Agilent Technologies, Santa Clara, CA, USA) using a Prodigy ODS(2) column (150 × 3.2 mm; 5 μm particle size) (Phenomenex, Torrance, CA, USA) with a 33 min nonlinear gradient of phase A (28 mM K_2_HPO_4_, 59 mM H_3_PO_4_) and phase B (75% phase A, 25% acetonitrile): 10% B (0−2 min); 10−20% B (2−4 min); 20−47% B (4−20 min); 47−80% B (20−25 min); 100% B (25−30 min); and 10% B (30−33 min) with a 1 ml min^−1^ flow. Folate derivatives were detected by a four-channel electrochemical detector (CoulArray Model 5600A, ESA, Massachusetts, USA) with potentials set at 100, 200, 300 and 400 mV. THF, 5-methyl-THF, 5,10-methenyl-THF and 5-formyl-THF were quantified using calibration curves made with standards obtained from Schircks (Schircks Laboratories, Buechstrasse, Jona Switzerland). Because of the acidic pH of the mobile phase, in these analyses, THF represents THF+5,10-methylene-THF and 5,10-CH=THF comprises 5,10-CH=THF+10-CHO-THF^33^.

### Root growth assays and global DNA methylation analyses

Seeds were germinated on Phyto agar (RPI Corp.) containing 1 × Murashige and Skoog Basalt Salt Mixture (MP) and 500 μM (6R,S)-5-CHO-5,6,7,8-THF calcium salt (Schircks Laboratories); 50, 100 or 250 μM L-methionine (SIGMA); 5 μM SMZ (SIGMA); 500 μM (6R,S)-5-CH3-5,6,7,8-THF calcium salt (Schircks Laboratories), which has been re-applied directly to the roots every 3 days due to its instability; or mock. Seedlings were grown vertically at 16 h light/ 8 h dark cycles and 22 °C. Root lengths were measured at 6, 8, 10, 12 and 14 days after germination and growth rates were calculated by linear regression from at least 10 seedlings per genotype per replicate. For measurement of global DNA methylation, seedlings were pooled per genotype and isolated genomic DNA was analysed by BS-seq as described above, except that EZ DNA Methylation-Lightning kit (Zymo Research) was used for BS conversion. DNA methylation levels were calculated as #C/(#C+#T) at CG, CHG and CHH sites and averaged over previously defined *mthfd1-1* DMRs. Read statistics are listed in Supplementary Table 3.

### Data availability statement

Primary high-throughput sequencing data that support the findings of this study have been deposited in the Gene Expression Omnibus (GEO) with the accession code GSE77966 (https://www.ncbi.nlm.nih.gov/geo/query/acc.cgi?acc=GSE77966). Secondary high-throughput sequencing data that support the findings of this study are available in the Gene Expression Omnibus (GEO) with the accession code GSE39901 (https://www.ncbi.nlm.nih.gov/geo/query/acc.cgi?acc=GSE39901). The authors declare that all other relevant data supporting the findings of this study and computer code are available within the article and its Supplementary Information files or on request.

## Additional information

**Accession codes:** All generated high-throughput sequencing data are available at NCBI's Gene Expression Omnibus (GEO) and are accessible via GEO Series accession number GSE77966.

**How to cite this article:** Groth, M. *et al.* MTHFD1 controls DNA methylation in *Arabidopsis*. *Nat. Commun.* 7:11640 doi: 10.1038/ncomms11640 (2016).

## Supplementary Material

Supplementary Information Supplementary Figures 1 - 10, Supplementary Tables 1 - 3 and Supplementary References 1 - 3

Supplementary Data 1 TEs differentialy expressed in mthfd1-1

Supplementary Data 2 PCGs differentialy expressed in mthfd1-1
