# Supplementary material for: MTHFD1 controls DNA methylation in Arabidopsis
Source: Nat Commun. 2016 Jun 13;7:11640. doi: 10.1038/ncomms11640 (PMC4909953; doi:10.1038/ncomms11640)
Supplement: Supplementary Information — Supplementary Figures 1 - 10, Supplementary Tables 1 - 3 and Supplementary References 1 - 3 [file ncomms11640-s1.pdf]

Supplementary Figure 1

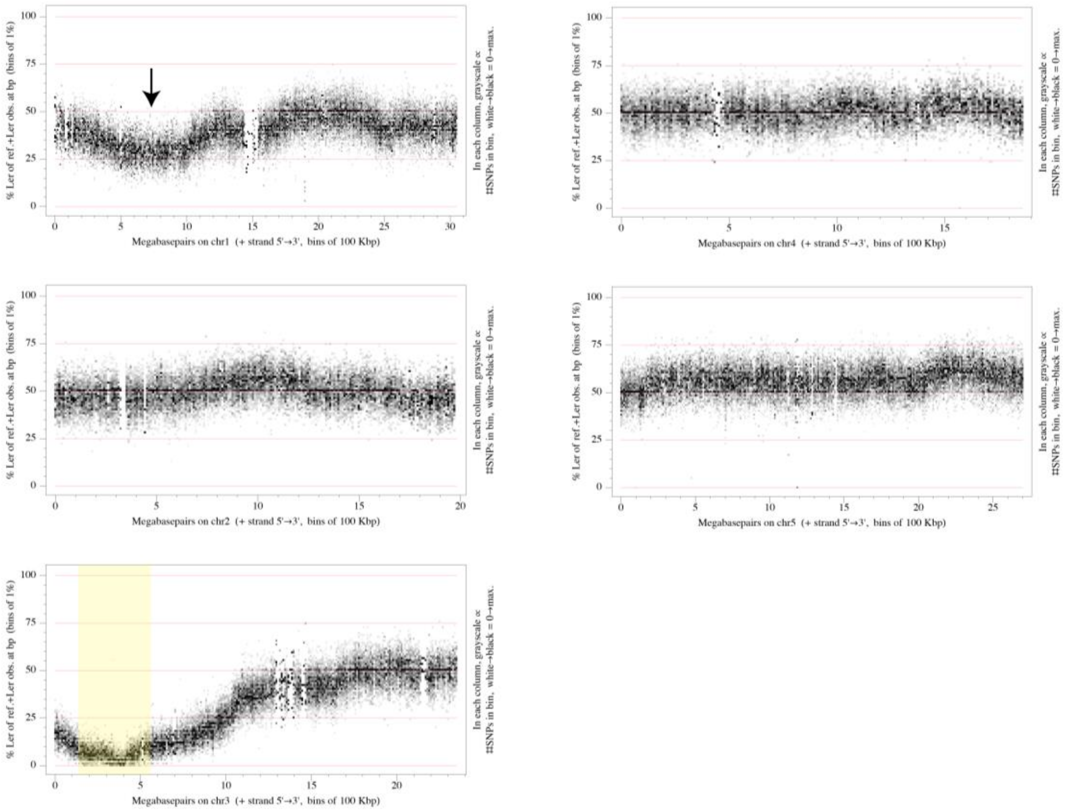

Supplementary Figure 1. Mapping of *mthfd1-1* by next-generation sequencing of pooled F2 mutants from a #162 x Ler cross.

Depletion of single nucleotide polymorphisms (SNPs) of *the* Landsberg (Ler) ecotype defines the target interval for the causative mutation in #162 (highlighted in yellow). The arrow indicates the insertion position of the *SDCpro-GFP* marker.

Supplementary Figure 2

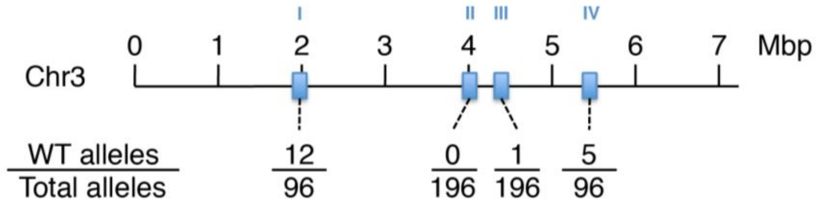

dCAPS marker	EMS polymorphism	Gene ID	Description
I	Chr3:01986370CtoT	AT3G06480	DEAD box RNA helicase
II	Chr3:03920848GtoA	AT3G12290	Tetrahydrofolate dehydrogenase/cyclohydrolase
III	Chr3:04381020GtoA	AT3G13445	TRANSCRIPTION FACTOR IID-1
IV	Chr3:05409109CtoT	AT3G15970	Ran-binding protein 1 domain-containing protein

Supplementary Figure 2. Fine mapping of *mthfd1-1.*

Co-segregation analysis of dCAPS markers from 4 candidate mutations in F2 mutants from a #162 x WT cross. Locations on chromosome 3 and allele ratios for each marker are indicated.

Supplementary Figure 3

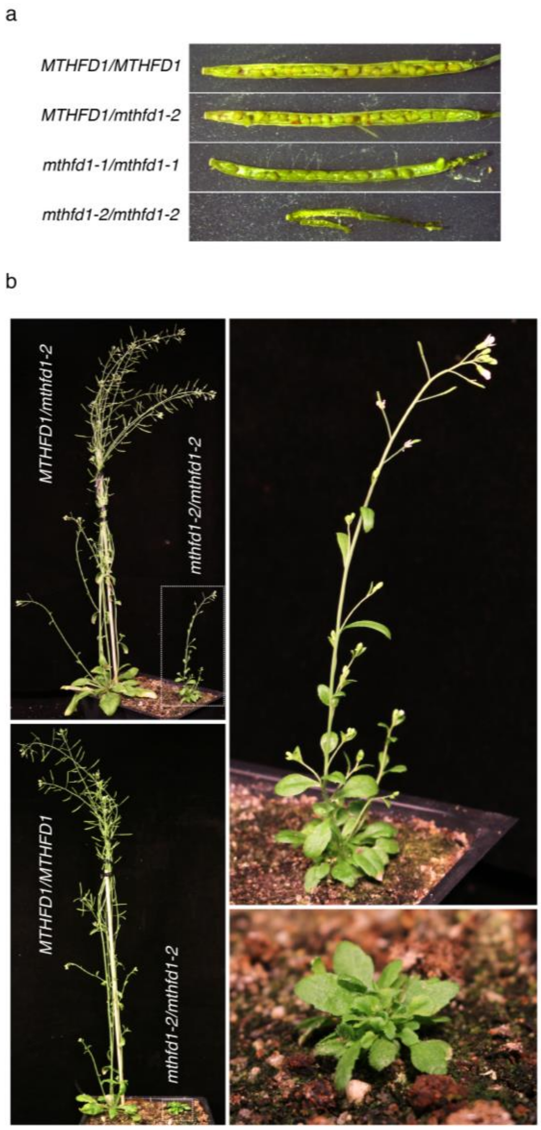

Supplementary Figure 3. Developmental phenotypes of *mthfd1* mutants.

(a) Siliques of homozygous *mthfd1-1* mutants show reduced number of seeds and homozygous *mthfd1-2* mutants are infertile.

(b) Wild-type and *mthfd1-2* mutant plants at 7 weeks after germination. Dashed boxes indicate close-ups of *mthfd1-2* mutants in right panels.

Supplementary Figure 4

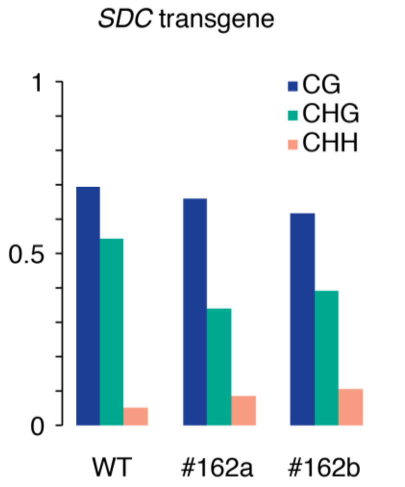

Supplementary Figure 4. Bisulfite sequencing of the repeat region of the transgenic *SDC* promoter.

DNA methylation levels for individual sequence contexts are shown and were calculated from at least 20 clones per sample.

Supplementary Figure 5

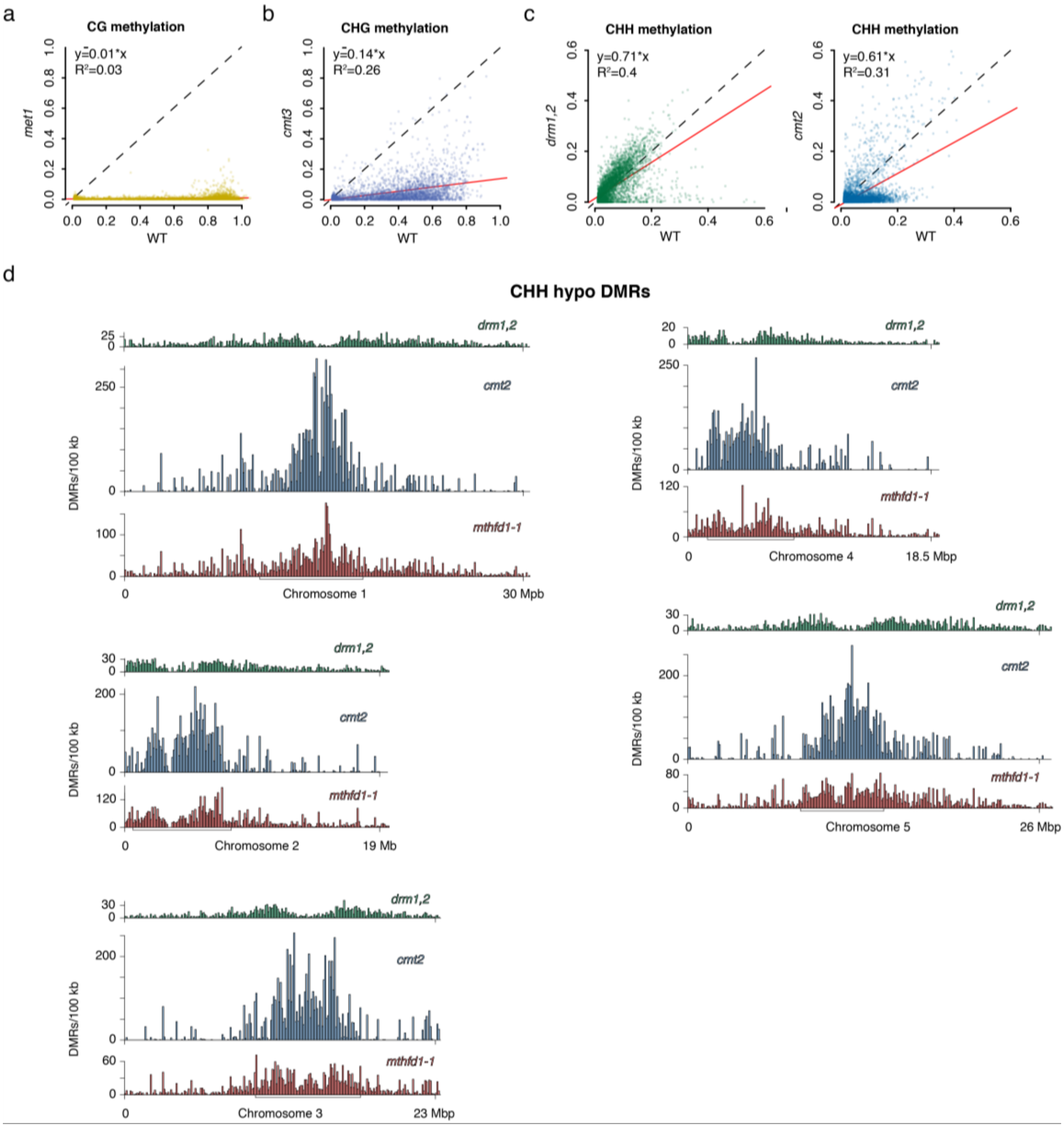

Supplementary Figure 5. Comparison of genome-wide DNA methylation patterns in *mthfd1-1* and DNA methyltransferase mutants.

(a-c) Comparison of DNA methylation levels in 5,000 random 100 bp bins with WT methylation levels > 0.01 in CG **(a)**, CHG **(b)**, and CHH **(c)** contexts. Red line: linear regression between mutant and WT levels; corresponding coefficients are shown in top left corners. Dashed: identity line. **(d)** Frequency distributions of CHH hypo DMRs in 100 kb bins along the chromosomes. Boxes indicate pericentromeric regions.

Supplementary Figure 6

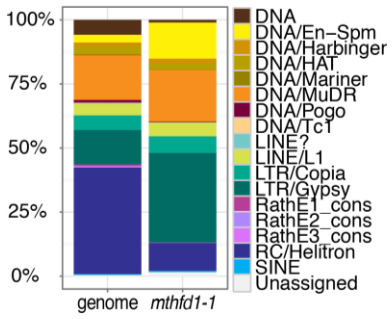

Supplementary Figure 6. Relative distribution of TEs in transposon superfamilies.

The distribution of TEs upregulated in *mthfd1-1* relative to WT is compared to the genomic distribution of all TEs.

Supplementary Figure 7

```
A2RVV7|MTHFD2_ARATH ------MLMIARKALASAHTKAFRLATRDVHCFSSILVSPPLVSLDLPENWIPYSDPPPP 56
Q9LHH7|MTHFD1_ARATH ------------------------------------------------------------
O65269|FOLD3_ARATH  ----MFTDCSSSTTSRLIHLYNRNGVFLPRPSVSQFSLRTTASTWRCTLSIRS------- 54
O65271|FOLD4_ARATH  MASMMFTDCSSTTTSRLIHLNRSSGTFLLRQCVGQLRLQTTASGRGCCIRSSSSPISSIS 60

A2RVV7|MTHFD2_ARATH VSFETEQKTVVIDGNVIAEEIRTKIISEVGKMKKAVGKVPGLAVVLVGEQRDSQTYVRNK 114
Q9LHH7|MTHFD1_ARATH MASSSDHTAKIIDGKAIAHTIRSEIAEEVRGLSEKHGKVPGLAVVIVGSRKDSQTYVNTK 60
O65269|FOLD3_ARATH  ---SSSPSAIVIDGKAEAKKIRDDIKIEVSRMKESIGVVPA------------------- 92
O65271|FOLD4_ARATH  ADTKSEGGAIVIDGKAVAKKIRDEITIEVSRMKESIGVIPGLAVILVGDRKDSATYVRNK 120

A2RVV7|MTHFD2_ARATH IKACEETGIKSVLAELPEDCTEGQIISVLRKFNEDTSIHGILVQLPLPQHLNESKILNMV 174
Q9LHH7|MTHFD1_ARATH RKACAEVGIKSFDVGLPEEVSEADLISKVHELNSNPDVHGILVQLPLPKHINEEHILGAI 120
O65269|FOLD3_ARATH  -----------------EDSSEEEVLKYVSGFNDDPSVHGVLVQLPLPSHMDEQNILNAV 130
O65271|FOLD4_ARATH  KKACDSVGIKSFEVRLAEDSSEEEVLKSVSGFNDDPSVHGILVQLPLPSHMDEQNILNAV 180

A2RVV7|MTHFD2_ARATH RLEKDVDGFHPLNVGNLAMRGREPLFVSCTPKGCVELLIRTGVEIAGKNAVVIGRSNIVG 234
Q9LHH7|MTHFD1_ARATH SIDKDVDGFHPLNIGKLAMKGREPLFLPCTPKGCLELLARSGVKIKGQRAVVVGRSNIVG 180
O65269|FOLD3_ARATH  SIEKDVDGFHPLNIGRLAMRGREPLFVPCTPKGCIELLHRYNIEFKGKRAVVIGRSNIVG 190
O65271|FOLD4_ARATH  SIEKDVDGFHPLNIGRLAMRGREPLFVPCTPKGCIELLHRYNIEIKGKRAVVIGRSNIVG 240

A2RVV7|MTHFD2_ARATH LPMSLLLQRHDATVSTVHAFTKDPEHITRKADIVIAAAGIPNLVRGSWLKPGAVVIDVGT 294
Q9LHH7|MTHFD1_ARATH LPVSLLLLKADATVTTVHSHTKDPEAIIREADIVIAACGQAHMIKGNWIKPGAAVIDVGT 240
O65269|FOLD3_ARATH  MPAALLLQKEDATVSIIHSRTMNPEELTRQADILISAVGKPNMVRGSWIKPGAVLIDVGI 250
O65271|FOLD4_ARATH  MPAALLLQREDATVSIIHSRTKNPEEITREADIIISAVGQPNMVRGSWIKPGAVLIDVGI 300

A2RVV7|MTHFD2_ARATH TPVEDSSCEFGYRLVGDVCYEEALGVASAITPVPGGVGPMTITMLLCNTLEAAKRIFL-- 352
Q9LHH7|MTHFD1_ARATH NAVSDPSKKSGYRLVGDVDFAEASKVAGFITPVPGGVGPMTVAMLLRNTVDGAKRVFGE- 299
O65269|FOLD3_ARATH  KPVEDPSAAGGERLVGDICYVEASKIASAITPVPGDVGPMTIAMLLSNTLTSAKRIHNFQ 310
O65271|FOLD4_ARATH  NPVEDPSAARGYRLVGDICYEEASKVASAITPVPGGVGPMTIAMLLSNTLTSAKRIHNFQ 360
```

Supplementary Figure 7. Multiple sequence alignment of the four MTHFD1 homologs in *Arabidopsis.* Mitochondrial and plastidic targeting peptides are highlighted in bold and underlined, respectively [1-3]. Uniprot identifiers are shown at the beginning of each line.

Supplementary Figure 8

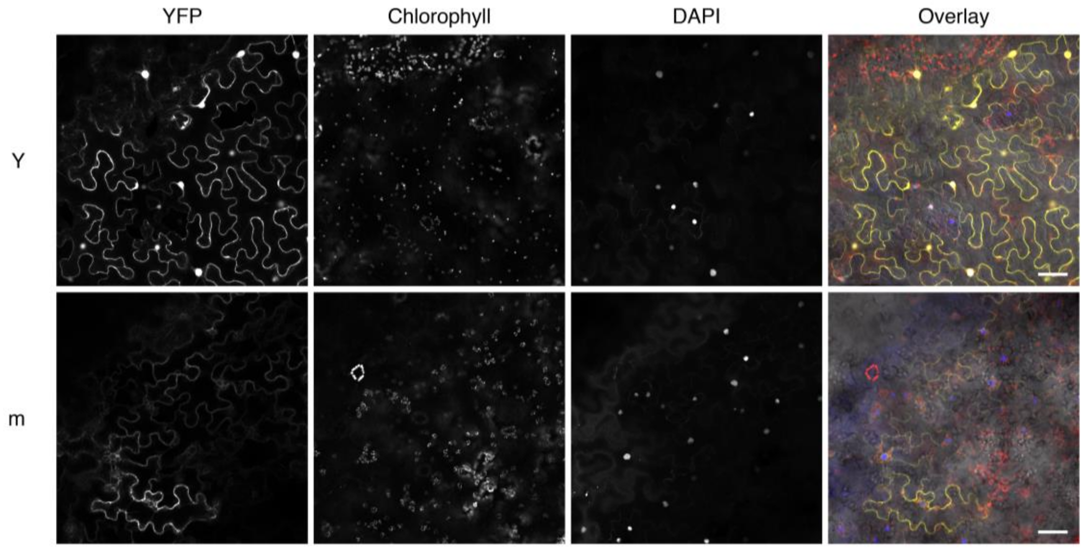

Supplementary Figure 8. Cytoplasmic localization of MTHFD1_R175Q-YPET-3xFLAG. Confocal micrographs of free YFP (Y) and MTHFD1_R175Q-YPET-3xFLAG (m) transiently expressed in *N. benthamiana*. Excitation (λ, nm)/Filter (λ, nm): YFP = 514/519-559, Chlorophyll = 488/630-730, DAPI = 405/409-530, and fluorescence overlay with bright field. Scale bars: 50 µm.

Supplementary Figure 9

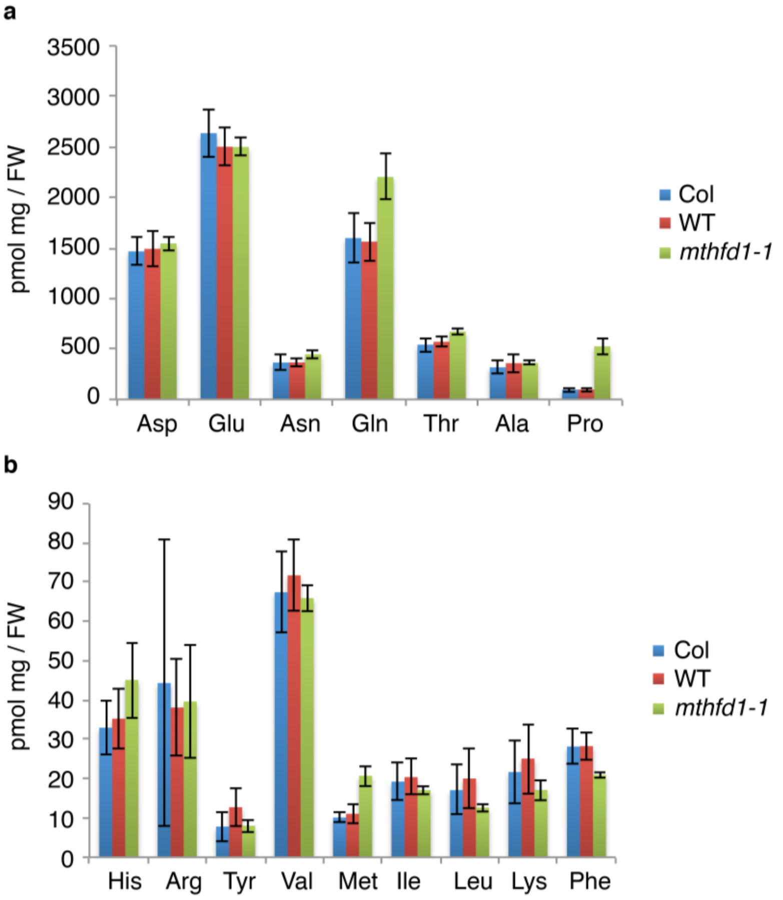

Supplementary Figure 9. Amino acid levels in rosette leaves of wild type plants without (Col) and with *SDCpro-GFP* (WT) and *mthfd1-1* mutants 3 weeks after germination.

Mean steady state levels ± SD (n≥3) for highly (**a**) and lowly abundant (**b**) amino acids are shown.

Supplementary Figure 10

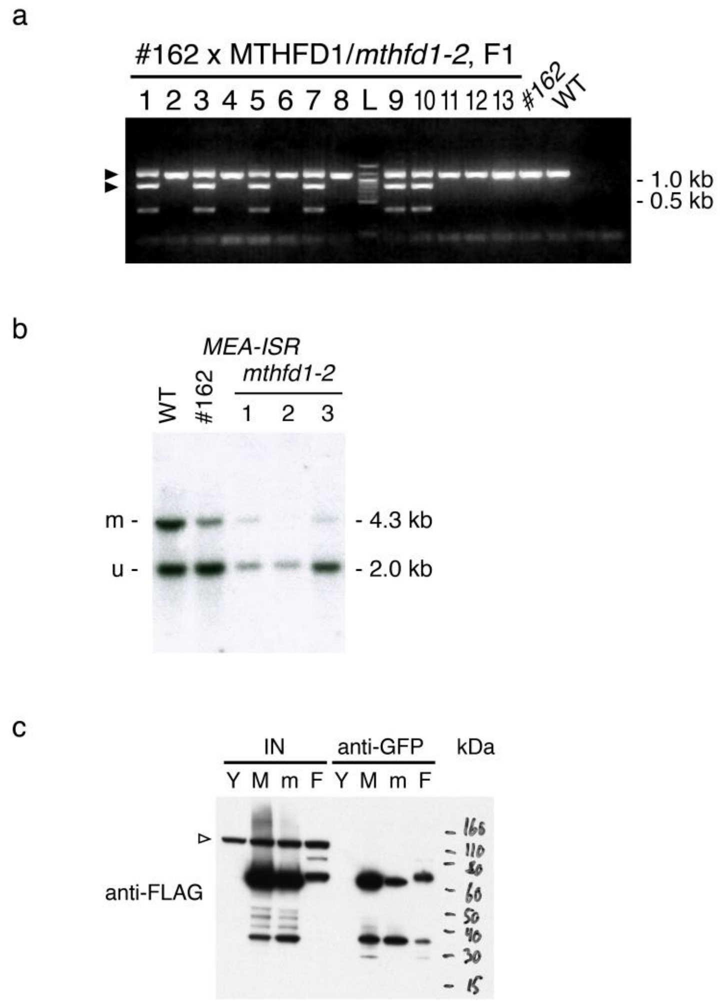

Supplementary Figure 10. Uncropped gel and blot images. **(a)** Uncropped image of ethidium bromide agarose gel shown in main Figure 1c. Arrowheads mark bands corresponding to WT/*mthfd1-1* (upper) and *mthfd1-2* (lower). **(b)** Uncropped image of DNA blot shown in main Figure 1e. Upper and lower bands correspond to methylated (m) and unmethylated (u) fragments, respectively. **(c)** Uncropped image of Western blot shown in main Figure 6b. Free YFP (Y), MTHFD1-YPET-3xFLAG (M), MTHFD1_R175Q- YPET-3xFLAG (m), or FOLD4-YPET-3xFLAG (F). Arrowhead indicates unspecific binding of anti-FLAG (shown as loading reference).

Supplementary Table 1. Survival of *mthfd1-2* mutants 3 weeks after planting

Parent	# wild-type	# mutants	# of seeds planted
A	81	7	102
B	51	3	66
C	27	1	30
Total	159	11	198

Supplementary Table 2. Primers used in this work.

Co-segregation analysis

Name	Sequence (5'-3')	Description
JP10089	CAACGAGCAGTTGTTGTAGGCC	CAPS II fo, digest PCR product with HpaII
JP10090	TGGTGTGAGAATGTACAGTTGTG	CAPS II re, digest PCR product with HpaII
JP10093	GGTTAAAGATAAAAAAAATCTACCTTA CTAG	CAPS III fo, digest PCR product with SpeI
JP10094	GTTTTATGTTTCCAGCGTTTTG	CAPS III re, digest PCR product with SpeI
JP10038	ACCAAAGCCTCTACCACTACCAC	CAPS I fo, digest PCR product with AluI
JP10039	TGGCCGTGAGGGTGGGTTTGGAAGC	CAPS I re, digest PCR product with AluI
JP9576	AGCATCTTCAGCACTCGGT	CAPS IV fo, digest PCR product with HinfI
JP9580	CAGGTTGACCTTAAACGCAAG	CAPS IV re, digest PCR product with HinfI

Genotyping of T-DNA insertion mutants

Name	Sequence (5'-3')	Description
JP10325	GCGGGAAATTACATATTTGCC	WiscDsLox244C04_LP
JP10326	TGCACCCAATATATGCTCCTC	WiscDsLox244C04_RP
JP10327	ACAATGTCAGCTTCCCGTATG	SALK_015165.35.05.x_LP
JP10328	GCGGTCTATCTGAGAAACACG	SALK_015165.35.05.x_RP
JP10203	TTCCAACATCAATTACTGCAGC	SALK_039538_LP
JP10204	CACTGTTATTTTGTTTCGCTGG	SALK_039538_RP

Bisulfite sequencing

Name	Sequence (5'-3')	Description
JP6349	GAAAAAGTTGGAATGGGTTTGGAGAG TTTAA	SDC_BSPCR fo
JP6350	CAACAAACCCTAATATATTTTATATTAA AAC	SDC_BSPCR re

Chop-PCR

Name	Sequence (5'-3')	Description
JP6699	ACTTAATTAGCACTCAAATTAAACAAAA TAAGT	*AtSN1* fo
JP6700	TTTAAACATAAGAAGAAGTTCCTTTTTC ATCTAC	*AtSN1* re

DNA blot

Name	Sequence (5'-3')	Description
JP980	AAACCTTTCGTAAGCTACAGCCACTTT GTT	*MEA-ISR* fo
JP981	TCGGATTGGTTCTTCCTACCTCTTTAC CTT	*MEA-ISR* re

Real-time RT-PCR

Name	Sequence (5'-3')	Description
JP9642	GTATCCTTTGGCCCGGTATT	ROMANIAT5 fo
JP9643	GCCTCTTCGAAATGCCATAA	ROMANIAT5 re
JP9055	AGTCCTTTTGGTTGCTGAACA	ATCOPIA28 fo
JP9056	CCGGATGTAGCAACATTCACT	ATCOPIA28 re
JP9640	AACTAACGTCATTACATACACATCTTG	soloLTR fo
JP9641	AATTAGGATCTTGTTTGCCAGCTA	soloLTR re
JP10137	TATGTTTGCGGTGGGTGCTGTG	SADHU3-2 fo
JP10138	ACAGCCTAAACCCACCAATCCG	SADHU3-2 re
JP2452	TCGTGGTGGTGAGTTTGTTAC	ACTIN fo

JP2453	CAGCATCATCACAAGCATCC	ACTIN re

Molecular cloning

Name	Sequence (5'-3')	Description
JP14184	CATAGTCTCgAgCGCAGCTGAAAACATGC	Genomic At3g12290 plus XhoI fo
JP14185	AGTAGAactagTccCTCGCCAAAGACACGC	Genomic At3g12290 plus SpeI re
JP14186	CTTCGTCATCTTCGAGTGTGAGT	Sequencing of pMLBART-MTHFD1-YPET-3xFLAG
JP14187	ATGTCCATGGTTAGTTGTGGCGG	
JP14188	GATCCTGAGGCTATCATACGG	
JP14189	GGATGGGCACCACGCCGGTGAACAG	
JP14190	TTTTTgTCGacTCTGGTAAGACCACACAATTTCAAC	Genomic At4g00620 plus SalI fo
JP14191	AAGTGACtaGTCcCTGGAAGTTGTGAATCCTCTTAGC	Genomic At4g00620 plus SpeI re
JP14192	CATCGTCTCTTCTCTGCTCTGC	Sequencing of pMLBART-FOLD4-YPET-3xFLAG
JP14193	AGCAGTAATCCTTGTTGGTGACAG	
JP14194	GATGCAACCGTTAGCATTATCC	

Supplementary Table 3. Next-generation sequencing reads statistics.

Whole genome bisulfite sequencing libraries	Sample	Total reads	Uniquely mapping	Chloroplast DNA methylation
SxaQSEQsXB015L2	WT	198,893,778	170,067,128 (85.5%)	CG: 0.00467, CHG: 0.0043, CHH: 0.00342
SxaQSEQsXB015L3	*mthfd1-1*	209,247,195	171,879,530 (82.1%)	CG: 0.00353, CHG: 0.00318, CHH: 0.00254
Libraries for global DNA methylation analysis	Sample	Total reads	Uniquely mapping	Chloroplast DNA methylation
SxaQSEQsVA089L8_idx13	WT mock, rep. 1	10,125,290	7,623,663 (75.3%)	CG: 0.00365, CHG: 0.00358, CHH: 0.00361
SxaQSEQsVA089L8_idx14	WT mock, rep. 2	8,836,836	7,735,717 (87.5%)	CG: 0.00333, CHG: 0.00323, CHH: 0.00334
SxaQSEQsVA089L8_idx15	mthfd1-1 mock, rep. 1	9,942,581	8,578,233 (86.3%)	CG: 0.00355, CHG: 0.00323, CHH: 0.00354
SxaQSEQsVA089L8_idx16	mthfd1-1 mock, rep. 2	8,411,130	6,340,794 (75.4%)	CG: 0.00367, CHG: 0.00334, CHH: 0.00343
SxaQSEQsVA089L8_idx18	WT 5-CHO-THF, rep. 1	9,530,658	7,895,562 (82.8%)	CG: 0.00311, CHG: 0.00297, CHH: 0.00315
SxaQSEQsVA089L8_idx19	WT 5-CHO-THF, rep. 2	8,168,179	6,031,979 (73.8%)	CG: 0.00319, CHG: 0.00289, CHH: 0.00302
SxaQSEQsVA089L8_idx20	mthfd1-1 5-CHO-THF, rep. 1	9,315,578	5,901,462 (63.4%)	CG: 0.00331, CHG: 0.00303, CHH: 0.00308
SxaQSEQsVA089L8_idx21	mthfd1-1 5-CHO-THF, rep. 2	9,912,881	7,821,124 (78.9%)	CG: 0.00331, CHG: 0.0029, CHH: 0.00326
SxaQSEQsVA089L8_idx05	WT Met., rep. 1	10,184,219	8,563,684 (84.1%)	CG: 0.00317, CHG: 0.00298, CHH: 0.00304
SxaQSEQsVA089L8_idx06	WT Met, rep. 2	11,717,668	8,575,529 (73.2%)	CG: 0.00333, CHG: 0.00298, CHH: 0.0031
SxaQSEQsVA089L8_idx09	mthfd1-1 Met, rep. 1	8,136,220	5,558,156 (68.3%)	CG: 0.0034, CHG: 0.00296, CHH: 0.00341
SxaQSEQsVA089L8_idx12	mthfd1-1 Met, rep. 2	9,643,906	6,655,212 (69.0%)	CG: 0.00321, CHG: 0.00298, CHH: 0.00326
SxaQSEQsVA089L8_idx25	WT SMZ	9,811,942	8,260,623 (84.2%)	CG: 0.00309, CHG: 0.00325, CHH: 0.0033
SxaQSEQsVA089L8_idx27	mthfd1-1 SMZ	11,300,244	7,895,026 (69.9%)	CG: 0.00323, CHG: 0.00288, CHH: 0.00315
SxaQSEQsWA135L8_idx_22	WT 5-CH3-THF, rep. 1	12,106,226	10,810,330 (89.3%)	CG: 0.00474, CHG: 0.00441, CHH: 0.00510
SxaQSEQsWA135L8_idx_23	WT 5-CH3-THF, rep. 2	12,843,726	11,106,325 (86.5%)	CG: 0.00400, CHG: 0.00389, CHH: 0.00407

SxaQSEQsWA135L8_idx_25	mthfd1-1 5-CH3-THF, rep. 1	14,776,761	12,983,150 (87.9%)	CG: 0.00392, CHG: 0.00372, CHH: 0.00403
SxaQSEQsWA135L8_idx_27	mthfd1-1 5-CH3-THF, rep. 2	15,142,392	13,010,480 (85.9%)	CG: 0.00391, CHG: 0.00372, CHH: 0.00401
RNA-seq libraries	Sample	Total reads	Mapping	Mapping with >1 and <21 alignments
SxaQSEQsXA013L5_idx5	*mthfd1-1 a*	24,452,864	23,105,348 (94.5%)	1,077,215
SxaQSEQsXA013L5_idx7	*mthfd1-1 b*	22,024,389	20,798,917 (94.4%)	828,238
SxaQSEQsXA013L5_idx9	WT	19,750,105	18,613,116 (94.2%)	849,450

Supplementary References

1 Collakova, E. *et al.* Arabidopsis 10-formyl tetrahydrofolate deformylases are essential for photorespiration. *Plant Cell* **20**, 1818-1832, doi:10.1105/tpc.108.058701 (2008).

2 Ito, J. *et al.* Analysis of the Arabidopsis cytosolic proteome highlights subcellular partitioning of central plant metabolism. *J Proteome Res* **10**, 1571-1582, doi:10.1021/pr1009433 (2011).

3 Zybailov, B. *et al.* Sorting signals, N-terminal modifications and abundance of the chloroplast proteome. *PLoS One* **3**, e1994, doi:10.1371/journal.pone.0001994 (2008).
